# Carbon Fiber Reinforced Thermoplastics: From Materials to Manufacturing and Applications

**DOI:** 10.1002/adma.202418709

**Published:** 2025-04-22

**Authors:** Howard (Hao) Wang, Siqi Huo, Venkata Chevali, Wayne Hall, Arnt Offringa, Pingan Song, Hao Wang

**Affiliations:** ^1^ Centre for Future Materials University of Southern Queensland Springfield 4300 Australia; ^2^ Aachen Center for Integrative Lightweight Production of RWTH Aachen University (AZL) RWTH Aachen University 52074 Aachen Germany; ^3^ School of Engineering and Built Environment Griffith University Southport 4215 Australia; ^4^ GKN Aerospace Papendrecht 3351 Netherlands

**Keywords:** applications, automated manufacturing, carbon fiber, composites, thermoplastics

## Abstract

Carbon fiber reinforced thermoplastics (CFRTs) have witnessed a resurgence in recent times since their first industrial use over five decades ago, with revitalized interest from aerospace companies and other key sectors including energy and automotive sectors. CFRTs are increasingly gaining footholds in high volume rapid manufacturing in aerospace and nonaerospace sectors owing to their inherent recyclability. However, there has been a lack of contemporary and critical review on this topic so far. This work aims to review the recent advances in CFRTs with emphasis on high performance thermoplastics. Both continuous and discontinuous fiber forms in tape, organosheet and short/long fiber architectures are discussed, their processing and postprocessing methods are reviewed, with emphasis on state‐of‐the‐art welding techniques. Typical applications of CFRTs in industry are examined, including fuselage, pressure vessel, and automotive components. Finally, insights are provided into ongoing challenges, future development, and a roadmap for expediting the development of high‐performance CFRTs.

## Graphical abstract

A contemporary and critical review on carbon fiber reinforced thermoplastics. The common thermoplastic and carbon fiber applied in the manufacture of carbon fiber reinforced thermoplastics are summarized, and the processing and postprocessing methods are reviewed, with emphasis on state‐of‐the‐art welding techniques. The typical applications of carbon fiber reinforced thermoplastics in industry are also highlighted.

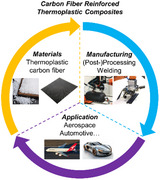

## 1 Introduction

Carbon fiber reinforced polymers (CFRPs) are advanced materials consisting of carbon fiber infused with a thermoset or thermoplastic polymer matrix, which possess high modulus and strength, and suitable for end uses which necessitate high strength, and stiffness at a low weight.^[^ ^1^ ^]^ Over the last five decades, CFRPs have been used predominantly in aerospace, and advanced engineering applications such as automobile, rail transportation, sports, leisure equipment, marine, and more recently, wind energy.

CFRPs in the aerospace industry have gradually displaced the predominant aluminum alloys through critical weight savings, with Boeing using 50 wt% of these materials in airframe and primary structures of Boeing 787 Dreamliner (B787) while overall aluminum fraction decreasing to 20 wt%, resulting in up to 22% fuel savings. The Airbus A350 platform also used CFRP (up to 53 wt%), simultaneously realizing 50% lower maintenance of structures and the airframe, hence extending the service interval substantially over its predecessor, A380. The adoption of CFRP in aerospace has accelerated over the last 4 decades, with increasing CFRP fraction attributed to the increased fuel efficiency, lowering carbon dioxide (CO_2_) emissions, reducing maintenance costs, and achieving higher design freedom through integration of parts. This desirable range of attributes associated with CFRPs have led to the production of over 9000 aircrafts the next two decades, with light aircraft using up to 70–80 wt% CFRPs. This burst of rapid adoption of CFRPs in aerospace is often termed as the first wave of CFRP applications.^[^ ^1^, ^2^ ^]^

The second wave of CFRP applications was witnessed via the expansion into nonaerospace, industrial end uses, which are characterized as large volume and low‐cost.^[^ ^1^ ^]^ This development of CFRPs is also accompanied by a rapid expansion of the carbon fiber production volume, capacity, and cost reduction. One example of this is wind blades, such as Vestas using CFRPs in turbine blades exceeding 100 m in length at an estimated 38% reduction in mass and 14% reduction in cost, in addition to increased capacity and power generation. This large volume application demand influenced the production capacity of carbon fiber and today, the wind energy sector uses more carbon fiber for wind turbine blades than the aerospace sector.

The nonaerospace utilization of CFRPs has ultimately led to production of carbon fibers with new technologies such as a large tow.^[^ ^2^ ^]^ Alternate precursors materials and modified conversion have driven the cost of carbon fiber down in the last 2 decades. The combination of enhanced properties and lowered cost of carbon fibers have resulted in addressing key bottlenecks for carbon fiber usage for advanced composites, which opens new CFRP applications in wind energy, automobile, rail, light aviation, building and construction, and other key industries.^[^ ^3^ ^]^

Currently, the overwhelming majority of CFRPs are thermosetting resin based composites with epoxies leading the market.^[^ ^4^, ^5^ ^]^ As polymer matrixes in CFRPs, thermosetting resins feature great mechanical properties, chemical resistance, and thermal/dimensional stability due to their three‐dimensional cross‐linked networks.^[^ ^6^, ^7^, ^8^ ^]^ However, the cross‐linked networks make the thermosets difficult to degrade and recycle, and thus the end‐of‐life (EoL) carbon fiber reinforced thermosets can only be processed by landfill, grinding, etc., which poses a great threat to the environment.^[^ ^9^ ^]^ Constructing covalent adaptable networks (CANs) within thermosets by introducing dynamic covalent bonds (DCBs) is an emerging approach to achieve the recycling of carbon fiber reinforced thermosets.^[^ ^10^, ^11^ ^]^ The common DCBs include imine bonds, ester bonds, disulfide bonds, and so on.^[^ ^12^, ^13^, ^14^ ^]^ Although CAN‐based thermosets can be recycled by physical or chemical methods, their mechanical properties and stability are subject to great doubts and they are therefore still far from commercialization.

Thermoplastic composites on the other hand possess unique attributes over both metallic alloys and thermoset composites.^[^ ^15^, ^16^ ^]^ Compared with metals, they possess higher stiffness‐to‐weight and strength‐to‐weight ratios, greater fatigue and corrosion resistance, and higher design freedom.^[^ ^17^ ^]^ CFRTs are made by combining carbon fiber reinforcement with thermoplastics, resulting in high mechanical strength and stiffness, good thermal stability, and high impact resistance. Compared with carbon‐fiber‐reinforced thermosets, CFRTs possess high toughness, low flammability, hygrothermal resistance, and inherently are recyclable and the feedstock can be stored for extended periods.^[^ ^1^, ^2^, ^18^, ^19^, ^20^ ^]^ CFRTs also allow substantially lower fabrication times and hence are suitable for high production rates at low cost.^[^ ^21^ ^]^ Furthermore, thermoplastics offer unique assembly options with dissimilar materials because of their high ease of formability.

For this reason, CFRTs have become increasingly popular in recent years.^[^ ^2^ ^]^ CFRTs possess a range of advantages over their thermoset counterparts and resultantly witness increasing interest in their applications. In the aerospace industry, these composites are used for structural components, such as wings, fuselages, and tail booms, because of their high strength and low weight. In the automotive industry, CFRTs are used for body and chassis components, as well as for interior trim and engine components. In the sporting goods industry, CFRTs are used for high‐performance bicycles, golf clubs, fishing rods and skis. In the industrial sector, they are used for structural components in corrosive environments, such as pipelines and offshore platforms. Research and development of CFRTs are surging, with a focus on improving the properties and performance of these advanced materials, exploring their potential within novel applications.

This review involves a critique of the recent progress in CFRTs, particularly in the last 5 years with a massive increase in published papers. The review starts with an introduction and history of CFRTs and goes on to discuss the main thermoplastic matrices and the forms of carbon fiber relevant for commercial CFRT applications. Then the processing and postprocessing techniques are elaborated. Welding of CFRTs, which is barely touched upon by other contemporary reviews, is discussed. With this understanding, the review then focuses on key applications and case studies that are relevant both from an academic and an industrial standpoint.

## 2 Development of CFRTs – A Historical Perspective

CFRPs have gained a strong foothold in the aerospace industry, with prime examples of B787 and A350 platforms utilizing over 50 wt% of CFRPs.^[^ ^2^ ^]^ However, the increasingly diverse and rapidly evolving CFRP markets necessitate large volume, high‐rate manufacturing at a low cost, beyond what carbon‐fiber‐reinforced thermosets can offer. Interest in CFRTs surged due to their potential for significantly greater fracture toughness compared to carbon‐fiber‐reinforced thermosets. Enhancing resin toughness was recognized as a key strategy to improve delamination resistance and damage tolerance in composites, prompting extensive research efforts globally. Furthermore, analysis tools originally created for carbon‐fiber‐reinforced thermosets, including cohesive zone models, fracture mechanics models such as virtual crack closure technique, and progressive damage models, were effectively applied on CFRTs without significant modifications. With the continuous development of CFRTs and technological shift from manual methods to automated manufacturing using preforms, innovative manufacturing techniques are well positioned to cater to the demands of the at‐scale manufacturing at higher rates for components with higher structural demand.

There has been a tremendous increase in the use of CFRTs in aerospace and automotive industries due to their clear advantages over carbon‐fiber‐reinforced thermosets.^[^ ^20^ ^]^ For instance, CFRTs replace metals such as aluminum, titanium, and steel alloys with clear benefits in weight savings (≈60%), increased specific strength (five‐fold), specific stiffness (two‐fold), greater fatigue performance and endurance (four‐fold), and the freedom to consolidation out of autoclave (OOA) and to obtain a higher level of design. Novel CFRTs are gaining increased attention compared to carbon‐fiber‐reinforced thermosets recently, because of their lower storage requirements and stability at room temperature. Furthermore, the OOA processing provides the opportunity to achieve shorter manufacturing cycles, ultimately requiring lower energy. CFRTs are readily recyclable, reformable, and reparable, which reduces a great deal of carbon emissions and keeps manufacturing sustainable.^[^ ^22^ ^]^ CFRTs utilization in aerospace is ever increasing with further growth forecasted.^[^ ^23^ ^]^

When we look at the history of CFRTs, we can date the roots of modern composites back to 1940s with the advent of the earliest composites patents.^[^ ^24^ ^]^ Since this time, thermoset polymer matrices such as epoxies have been the standard for use in composites, with thermoplastics being used relatively later in this timeline. In the 1960s and 1970s, thermoplastics were used in military and defense applications. During 1990s, many major chemical companies such as DuPont, Phillips Petrol, Exxon, and BASF shifted their focus back to thermoset composites. However, recent times have seen a resurgence in CFRT‐based developments, caused by environmental issues and appetite for faster production cycles. The increasing demand for high performance in the 1990s led DuPont, Phillips Petroleum and Exxon to divert their focus again towards synthesizing high performance thermoplastics driven by market demand and greater margins. These novel materials were used heavily in aerospace, automotive, and electronics. Eventually, the thermoplastics market became a highly competitive market which then opened exploration of diverse growth opportunities.

Today, the audacious net zero goals set by several nations are again beginning a revitalization and conversation of bringing back lightweight materials, where thermoplastics and CFRTs possess a distinct advantage.^[^ ^25^ ^]^ This is compounded by a move from use of metals to composites to achieving lightweighting of aircraft bodies. From a meagre 4% in the 1970s, composite use has now surpassed 50%, attesting to the advantage.^[^ ^2^ ^]^ Even today, CFRPs continue to be used predominantly in aircraft, and CFRTs are progressively being integrated into aircraft designs.

CFRTs show great processibility and recyclability, making them suitable for sustainable development. Compared with carbon‐fiber‐reinforced thermosets, CFRTs feature higher production rate with a shorter cycle time, automated assembly through welding, post processing and recycling through remelting, higher fracture toughness, and are more environmentally friendly. However, CFRTs have some disadvantages, such as requirements on high processing temperature and pressure, low cost‐efficiency, difficulty in impregnation, and poor chemical resistance.^[^ ^26^, ^27^ ^]^

CFRTs have received renewed interest in aerospace and have penetrated new markets in other industrial sectors because of the ability to be automated to produce low‐porosity consolidated structures and to be fusion welded to reduce assembly, making them the preferred choice. CFRTs overall have now found use in nacelles, doors, brackets, ribs, floor panels, wing leading edges, rudders, and elevators of aircraft. CFRTs are also used in flight control surfaces and offer 30% lower cost and 40% lower cycle times than metal.

## 3 Thermoplastics and Carbon Fibers in CFRTs

CFRTs are composed of a thermoplastic polymer matrix and carbon fibers, with an engineered interface consisting of a sizing or coating to create a pathway for load transfer from the thermoplastic matrix to the carbon fibers. The aerospace industry predominantly uses high performance thermoplastic polymers with melting points reaching up to 350 °C.^[^ ^2^, ^23^, ^24^, ^28^, ^29^ ^]^ However, certain engineering thermoplastics such as polyamides and even commodity thermoplastics such as polyolefins are often used in conjunction with carbon fibers in nonaerospace sectors. Polyolefins specifically are used in conjunction with discontinuous carbon fiber forms. In this section, the common thermoplastics and carbon fibers used in CFRTs are introduced, and the surface treatment methods of carbon fibers are explored, as they are critical to the performances of CFRTs.

### 3.1 Thermoplastic Matrix

**Figure 1:**
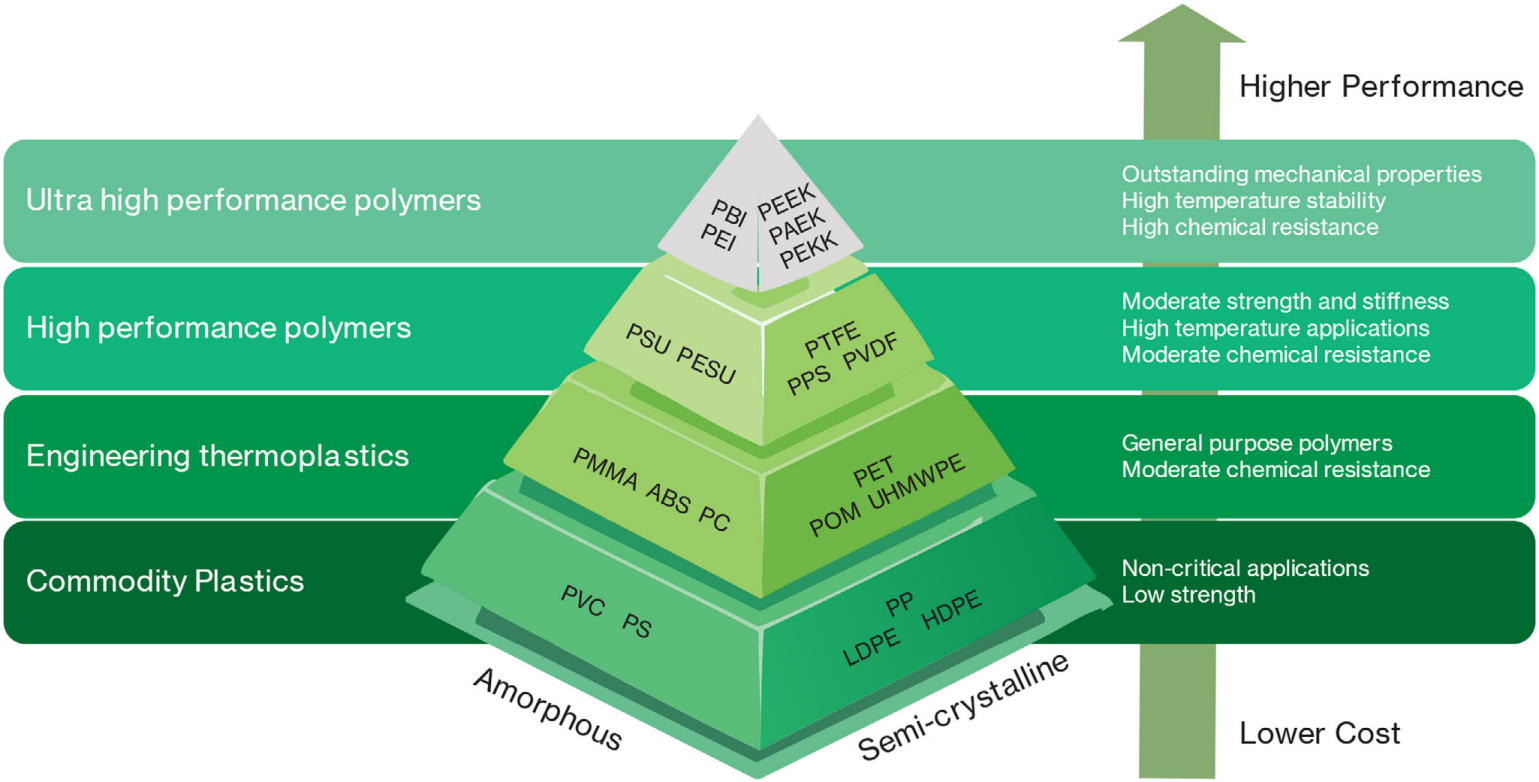
The hierarchy of amorphous and semicrystalline thermoplastics. Reproduced under the terms of the CC‐BY Creative Commons Attribution 4.0 International license (https://creativecommons.org/licenses/by/4.0).^[^ ^31^ ^]^ Copyright 2023, The Authors, published by MDPI.

The four major kinds of thermoplastic polymers are displayed in **Figure** **1**. Typically, higher performance polymers cost more and vice versa. Thermoplastic polymers are different from thermoset ones, and they can be repeatedly melted into a viscous liquid state in which shaping and forming can be achieved.^[^ ^30^ ^]^ They offer many opportunities for component manufacturing and joining as a result. Thermoplastic polymers, especially high‐performance ones including poly (ether ether ketone) (PEEK), were initially patented in 1978, and they gained prominence in the 1990s along with a diverse range of other thermoplastics for CFRTs. Thermoplastic polymers are traditionally and notoriously hard to process because of their high viscosity, which requires the application of high processing temperatures and pressures for forming and consolidation. Future development in CFRTs is heavily dependent on the coordination of processing, material and design, and it further necessitates vertical integration throughout the design cycle in the process of planning new developments. Poly (ether ether ketone) (PEEK), polyetherketoneketon (PEKK), low‐melt poly (aryl ether ketone) (LMPAEK), poly (phenylene sulfide) (PPS), poly (ether imide) (PEI), polyamide (PA), and polypropylene (PP) used as polymer matrix in CFRTs are introduced, and their costs and key performances are listed in **Table** **1**.

**Table 1:** Key properties of PEEK, PEKK, LMPAEK, PPS, PEI, PA, and PP.

Polymer	PEEK	PEKK	LMPAEK	PPS	PEI	PA	PP
*T*_g_ (°C)	143	150–165	120–130	85–95	215–225	50–70	−10–10
Crystallization temperature (°C)	280–300	260–290	250–280	225–275	–	180–240	110–130
*T*_m_ (°C)	343	305–360	270–290	280–290	–	210–270	160–180
Typical processing temperature (°C)	360–400	340–400	280–320	300–340	320–370	230–290	180–240
Cost (USD kg^−1^)	100–150	>200	150–200	5–15	15–25	2–5	2–5
Tensile strength (MPa)	90–100	90–100	90–100	80–90	100–105	80–95	30–40
Flexural modulus (GPa)	3.5–4.0	3.5–4.0	3.5–4.0	3.5–4.0	≈3.2	2.0–3.0	1.5–2.0
Notched Izod impact strength (J m^−1^)	80–90	80–90	80–90	70–80	50–60	50–100	20–30

#### 3.1.1 Poly (Ether Ether Ketone) (PEEK)

Within the polyaryletherketone family, different polymers exhibit distinct thermophysical properties, including glass transition temperature (*T*_g_) and melting temperature (*T*_m_), due to the difference in the mass ratio between ketone and ether.^[^ ^31^, ^32^, ^33^ ^]^ The form of carbon fiber, i.e., unidirectional or discontinuous dictates the mechanical properties of polyaryletherketone‐based CFRTs.^[^ ^34^ ^]^ The volume fraction of the fiber and the manufacturing method also further dictate the mechanical performance. The mechanical properties of polyaryletherketone‐based CFRTs are a function of the carbon fiber form, loading, and the manufacturing process. On a specific basis, the tensile performance of polyaryletherketones‐based CFRTs is superior to metallic alloys. Processing parameters have a considerable influence on the polyaryletherketone‐based CFRTs. PEEK exhibits a range of desirable mechanical properties, excellent fracture toughness, and exceptional thermal stability and chemical resistance (Table 1).^[^ ^35^ ^]^

#### 3.1.2 Polyetherketoneketon (PEKK)

Compared to PEEK, PEKK has a similar melting temperature but much lower crystallization temperature (Table 1), providing a wider process window. Thus, PEKK can stay longer than PEEK in molten status, delivering a lower crystallization speed and a better consolidation quality. However, crystallization of PEKK is more difficult than PEEK. The mechanical properties of PEKK are similar to those of PEEK (Table 1), but it provides better fire/smoke properties. PEKK exhibits exceptional toughness and good adhesion to other materials.^[^ ^36^ ^]^

#### 3.1.3 Low‐Melt Poly (Aryl Ether Ketone) (LMPAEK)

One of the ways to address the processing challenges of high‐performance polyaryletherketone polymers is the development and implementation of LMPAEK polymers, which require a lower forming temperature to obtain semifinished products.^[^ ^37^ ^]^ LMPAEK is a high‐performance polyaryletherketone for CFRTs, which offers many of the performance attributes of PEEK, but its melting temperature is about 50–70 °C lower than PEEK (Table 1).^[^ ^38^ ^]^ Unidirectional tape products with LMPAEK are revolutionizing the CFRT landscape with savings in costs related to infrastructure, setup, energy consumption, and part production. Furthermore, a broader processing window also brings further freedom and strength over comparable precursor materials.

#### 3.1.4 Poly (Phenylene Sulfide) (PPS)

PPS is a high‐performance thermoplastic polymer that offers thermal stability, flame retardance, and resistance to chemical corrosion, making it an ideal material for demanding environments.^[^ ^39^ ^]^ These advantages are somewhat diminished the brittleness of PPS (Table 1). PPS‐based composites are commonly used in protective coatings, chemical containers, building shell and automobile parts. PPS is often combined with carbon fibers complementing their superior mechanical strength, thermal conductivity, and low‐density hydrophobicity. PPS inherently presents a nonpolar, chemically‐inert surface because of the lack of available reactive functional groups, limiting its combination with carbon fiber. However, in comparison to PEEK, PPS is more cost‐effective.^[^ ^40^ ^]^

#### 3.1.5 Poly (Ether Imide) (PEI)

PEI, first synthesized in the 1980s under the trade name Ultem, is produced through the imidization of a diacid anhydride with m‐phenylene diamine.^[^ ^41^ ^]^ PEI exhibits a *T*_g_ of about 220 °C (Table 1) and is renowned for its exceptional heat resistance, rigidity, mechanical strength, electrical properties, flame resistance, and extensive chemical resistance.^[^ ^42^, ^43^ ^]^ PEI can be processed relatively with ease comparable to engineering thermoplastics, albeit higher *T*_m_ is required. PEI also endures multiple reprocessing cycles because of its high thermal stability. Because of a large processing window spanning over 100 °C, PEI can be processed using a broad range of processing methods, with complex geometries and dimensional stability in thin sections. PEI is often used in internal components of appliances, automobiles, and aerospace/transportation applications. PEI‐based CFRTs have witnessed a dramatic increase in usage in aerospace and commercial aviation. A limiting factor for the use of PEI‐based composites in structural applications, due to its amorphous nature, is the low chemical resistance against aggressive fluids, such as Skydrol hydraulic fuel, used in many airliners.

#### 3.1.6 Polyamide (PA)

PA with a common commercial name Nylon belongs to the engineering thermoplastics class and is commonly used in semistructural applications because of its rigidity and broad range of mechanical performance, which is often a substitute for traditional metallic alloys in a range of industrial applications.^[^ ^44^ ^]^ PA is semicrystalline in nature and often finds use in tribological applications such as gears. PA is a good candidate to be used as polymeric matrix in CFRTs because of its overall low cost and ease of handling (Table 1). Various polyamides are used with carbon fibers with the main types being Nylon 6 (PA6), Nylon 11 (PA11), Nylon 12 (PA12), and Nylon 6‐6 (PA6‐6). Nylon 6,6 (*T*_m_ = ≈265 °C) possesses a combination of physicochemical and mechanical properties that allows its use in several industries including aeronautics and makes it viable for space applications.

#### 3.1.7 Polypropylene (PP)

PP is a commodity thermoplastic with the largest production capacity and usage because of ease of processing, low cost, and high durability against a range of polar solvents and chemicals (Table 1).^[^ ^45^ ^]^ PP‐based CFRTs exhibits a challenging interface because of the hydrophobic nature of PP and the carbon fibers, which requires careful modification of the interface.^[^ ^46^ ^]^ Maleic anhydride in low loadings is often used to graft with PP and adhered to the carbon fiber surface to gain effective interfacial adhesion and resultantly, effective mechanical properties of the resultant CFRTs. The common fiber forms used with PP are discontinuous carbon fibers.

### 3.2 Carbon Fibers

Carbon fiber presents high specific strength and stiffness, which can be produced from polyacrylonitrile copolymers (PAN), pitch or natural resources such as cellulose. The market share of PAN‐based carbon fiber accounts for 96–98%.^[^ ^47^ ^]^ The natural resources are receiving increasing attention in carbon fiber production due to potentially lower carbon footprint and costs,^[^ ^47^, ^48^ ^]^ but the bio‐based carbon fiber suffers from poor yield and need for stretch‐graphitization.^[^ ^49^ ^]^ Thus, the carbon fibers introduced in this section are mainly PAN‐based.

Carbon fiber can be classified according to its fiber aspect ratio (length/diameter), and they are often either short‐fiber or long‐fiber, and continuous fiber.^[^ ^50^ ^]^ Continuous/discontinuous fiber aspect ratio (*l*/*d*) dictates the mechanical performance. The selection of processing method largely depends on the fiber length, along with processing parameters such as the process induced shear and temperatures chosen. Critical aspects such as fill, consolidation, fiber distribution, drapability around corners, fiber bridging, and delamination also are functions of fiber length and processing method. In short and long fibers, high shear processes such as injection molding are possible. In continuous carbon fiber, preforming, melt impregnation, prepreg, or commingling the fiber with polymer is preferred.^[^ ^51^ ^]^

#### 3.2.1 Continuous Carbon Fiber

##### Carbon Fiber Tape

A tape is a fully impregnated unidirectional fiber reinforced thermoplastic material with material thickness usually between 0.13 and 0.25 mm. On a continuous roll can reach up to 3000 m length and with a variable width which ranges from 2.5 mm to several 100 mm.^[^ ^52^ ^]^ The fiber volume fraction ranges from 40 to 60 wt%, with aerospace tapes usually lying in the range of 50% to 60% by weight. Common thermoplastic resins used to produce carbon fiber tapes include PP and PA (mainly for automotive applications) and PPS, PEEK, PEKK, and LMPAEK (mainly for aerospace applications).

Tapes are manufactured predominantly using four different techniques which include wet powder impregnation, dry powder impregnation, melt impregnation, film stacking, and in situ polymerization. Each of these manufacturing methods offers a distinctive advantage where wet powder impregnation provides a more consistent and even dispersion of the polymer while the dry powder impregnation process usually tends to create resin rich and resin deficient areas which can ultimately lead to nonuniformity and inter ply porosity. The tape architecture offers the best possible use for mechanical properties development because of the presence of predominantly unidirectional fibers in the product. However, this comes with a compromise in the fiber properties in the transverse direction because the fiber predominantly offers strength in the longitudinal direction. This furthermore brings upon an additional complexity in processing of the tapes because the waviness and other fiber related effects must be closely controlled.

Effective fiber wet out and homogeneous distribution are critical to achieve uniform impregnation of the polymer around the carbon fibers, specifically to avoid defects within the consolidated section and reduction of fiber driven defects such as fiber overlap, misalignment, waviness and, porosity. In various manufacturing processes, including film stacking, hot compaction, melting pultrusion, and both wet and dry powder impregnation, specific effects are typically observed. Among these, film stacking has gained significant prominence and has been employed for several decades in the production of fiber‐reinforced thermoplastic composites. This method involves laminating fibers between layers of thermoplastic films, which are then fused together under elevated temperatures and pressures, typically exceeding 10 bar, over a duration of approximately 60 min. The process creates a prepreg, however this can lead to damage of fibers and the impregnation of the fibers by the matrix which can bring about undesired effects. The creation of unidirectional fiber reinforced tapes using a more industrial processes such as pultrusion or dry impregnation are similar where the fiber tow is passed through a plastic polymer melt pool or similarly in a dry powder bed or polymer suspension to completely impregnated fiber with a thermoplastic present. Dry and wet processing methods typically necessitate a postheat treatment to melt the polymer and eliminate excess polymer. The impregnation of fibers with a thermoplastic melt, along with the stability of the thermoplastic polymer at room temperature, generally dictates the degree of overall consolidation. To some extent, a dry process usually precludes the resin viscosity related issues, however, a dry process also brings about additional friction between the fiber and the die which can often cause fiber or the tapes to be damaged or broken down. Electrostatic forces also interfere in a dry process, and often make it difficult to control the process with consistent fiber volume fraction. Wet consolidation utilizes surface tension, capillary forces, and powder distribution within the suspension, enabling enhanced control over consolidation. The presence of a liquid phase in this method notably reduces frictional forces between the tow and impregnation points, thereby minimizing fiber damage and facilitating the interlocking of powder with the tow upon exiting the bath.

The effect of materials and process selection steers the properties of the unidirectional tapes. On the matrix side, the viscosity and the melting temperature of the resin should be considered, and on the fiber side, the sizing, the fiber itself, and the fiber roving are critical. The impregnation unit has a significant effect on the quality of the unidirectional tape in melt impregnation process, where the fibers are impregnated using melt pool of the resin which flows parallel and perpendicular directions with respect to the fiber feed while the perpendicular part is more critical.^[^ ^53^, ^54^ ^]^

Thermoplastic resins solidify after recrystallizing below the melting point upon cooling through a natural draft of air or under pressure.^[^ ^55^ ^]^ After melt impregnation, a thermoplastic unidirectional (UD) tape is subjected to drawing in a high temperature oven which further re‐melts polymer to ensure homogeneous polymer impregnation, which helps to minimize porosity and to achieve the desired crystallinity in the consolidated matrix. The areal density of the tape is dictated by the balance between the tape width and the tape thickness. The evaporation of volatiles from the resin slurry is the key to producing thermoplastic tapes at a faster production rate. A global carbon manufacturer, Teijin, claims they can produce up to 320 tons per annum of thermoplastic tape based on their latest technological innovations in this area. The key quality criterion for thermoplastic unidirectional tape include a) tape width and thickness, b) surface reference, c) areal weight, d) fiber volume fraction, e) porosity, f) crystallinity, and g) fiber distribution, whereas mechanical properties are used for benchmarking against similar products.^[^ ^54^ ^]^ In aerospace, the thermoplastic unidirectional tapes such as CF‐PPS, CF‐PEEK, CF‐PEKK, and CF‐LMPAEK are mainly applied.^[^ ^28^ ^]^

LMPAEK is often thought of as an alternative to PEKK and PEEK for faster manufacturing. LMPAEK is effective in certain cases, such as automated fiber placement, stamp forming, and welding. Even though the lower melt often indicates the presence of low melting segments of polymers, which naturally indicates that the mechanical properties are lower. However, this is not the case with the LMPAEK, and moreover the cost is also competitive with respect to established carbon fiber thermoplastic tape variants such as the TC 1200 and TC 1320. In addition, LMPAEK has better flow properties over their traditional counterparts which also means that because of the higher speeds achievable the consolidation quality is also commensurate without the need for post processing.

Cetex CF/LMPAEK tapes such as Cetex TC1225 and TC 1320 are processable around 305–340 °C window. These tapes have been applied in fuselage demonstrators at thickness of a few mm, and often feature integrated strengthening such as ribs with local reinforcement. The elevated processing temperature of PEEK is often prohibitive when it comes to the moderation of process economics. Other polyaryletherketones are viable and offer acceptable levels of mechanical and fire‐smoke‐toxicity performance. These materials are currently used in the leading edges of the A380 aircraft. When compared with PPS, PEKK offers comparable processing window albeit with superior toughness and adhesion with dissimilar materials. These advantages, however, do not translate into true market shares because of the heavy competition and distinctions offered by the high performance of PEEK and high processability of LMPAEK.

In orthopedics, CF‐PP and CF‐polyethylene (PE) tapes are commonly utilized. In addition, CF‐polycarbonate (PC) tape is applied because of its excellent bonding properties. In automotive industry, CF‐PA6 and CF‐PP tapes are widely investigated with overmolding. For pressure vessels, CF‐PA12 tape is widely applied.

##### Organosheet

Organosheets are defined as fabric based semifinished products of CF with a thermoplastic polymer, fully impregnated and consolidated in the primary form. Typically, organosheets are made with aramid, glass, or carbon fibers, with matrices including PP, PA, PPS, PEEK, PEKK, and LMPAEK. They offer a shorter processing cycle time because of the completed impregnation in the primary, semifinished stage, which usually spans ≈60 s. The tensile and compressive behaviors, as expected, are dependent on the fiber orientation. Thermoforming is a common processing method applied on organosheets to obtain secondary or near finished products.^[^ ^55^ ^]^

Organosheet is produced using compression molding with fibers and a thermoplastic to achieve optimal impregnation at a low cost. Intrinsic high melt viscosity of thermoplastics precludes potential lowering of processing cost thereby opening further investigation into novel impregnation routes. Contemporary impregnation methods include a double belt press which features heat and pressure modules to implement impregnation of the polymer and then cooling and recrystallization, in an essentially continuous compression molding variant. The application of pressure is critical in organosheet processing, and a roller press is usually preferred in a double belt process over an isobaric press. During the processing, a pressure gradient develops along the outside roller and below, causing resin flow to occur simultaneously as the section is being consolidated.^[^ ^56^, ^57^ ^]^

Organosheet is commonly used in aeronautical, industrial, and automobile parts because of its potential of short cycle manufacturing using processes such as stamping and welding. A double belt process can be implemented as a continuous process, however, this requires heavy optimization and continually increasing knowledge of the impregnation mechanism. The main parameters to be optimized are the belt speeds pressure and temperature, which ultimately dictates the level of impregnation and thereby the mechanical properties of the organosheets.^[^ ^57^ ^]^ Consolidation entails applying pressure to the fiber preform to eliminate voids and porosity from the resin matrix, thereby establishing the precise fiber volume fraction. A matrix with greater viscosity necessitates higher pressure to attain a superior level of impregnation and consolidation. Even when the organosheets are provided in a preconsolidated form, they can lose this consolidation when heated above the melting temperature.^[^ ^55^ ^]^

Two‐dimensional (2D), fully‐impregnated and consolidated organosheets are typical semifinished products that must be heated prior to forming.^[^ ^55^ ^]^ Organosheets are used in conjunction with injection molding specifically for automotive processes for local reinforcement. One of the downsides of using organosheets with defined section thickness is the reduction in design freedom where the local stiffening requirement is not well defined.^[^ ^58^ ^]^

#### 3.2.2 Discontinuous Carbon Fiber

The short fiber format of carbon fiber is usually considered downstream from a continuous fiber application because short fiber usually is obtained as offcuts or after recycling of the EoL CFRPs. In this review, short fiber based CFRTs will be covered albeit not as the main focus.

It is well understood that with a steady growth of CFRPs, the EoL scenarios also grow in diversity. Waste management in the composite applications and in the composites manufacturing industry brings about novel challenges in the ecological impact, landfilling, and compliance with new legislation towards the responsible recycling or reuse of carbon fibers within CFRPs. A major avenue for the EoL management of carbon fibers is the application of waste carbon fibers as short, discontinuous fibers in thermoplastics, although they can be recombined with thermoset polymers as well. CFRTs with recycled carbon fibers offer several benefits in processing such as formability and facile consolidation, but more importantly reduce the overall impact as the embodied energy is reused. Furthermore, a 50% lower cost of recycled carbon fibers is a compelling case for use of recycled fibers as even with technological advancements, the future cost of virgin carbon fiber is anticipated to stay high.^[^ ^59^ ^]^ Discontinuous carbon fibers are reprocessed using injection molding, compression molding, and as nonwoven mats. In some cases, recycled carbon fibers are aligned unidirectionally using ultrasonic and immersion techniques to attain a high level of fiber alignment using short carbon fibers to regain a majority of lost mechanical performance in the consolidated form.^[^ ^18^ ^]^

##### Short Carbon Fiber

In short fiber‐based CFRTs, the fiber distribution often follows a random orientation and distribution within the thermoplastic matrix. Fiber loadings of 20–50 wt% are common, and the fiber related aspects are dictated by the processing method and design of the composite. Short fiber composites are usually compounded and supplied as pellets of 3–4 mm length, with fiber lengths in the 0.2–0.4 mm range. Short fiber composites are usually not recycled or reprocessed because of the deterioration of matrix and fiber properties during the repeated thermal cycling.^[^ ^60^ ^]^ Short fiber composites increasingly find application in welding of compression molded parts that are produced using unidirectional laminate skin. Furthermore, they have been used in butt joints that are common in ribs in aerospace. The use of short fiber composites in these specialized applications usually enhances the pull‐off strength of the ribs as the butt joint evenly distributes pull off loads.

##### Long Carbon Fiber

Long carbon fiber reinforced thermoplastics (LCFTs) are produced with virgin carbon fibers, and they can also be obtained from EoL CFRTs. Overall, the LCFTs are extruded and injection/compression molded into the final form using low shear extrusion (usually single screw).^[^ ^61^ ^]^ A high aspect ratio of carbon fiber in LCFTs endows them with great impact strength, high modulus, great dimensional stability, and strength,^[^ ^61^, ^62^ ^]^ because fiber type, length, and loading dictate the resultant properties. For LCFTs, the relatively long fiber length enhances adhesion with the matrix, forming a network structure of fibers. Consequently, the strength of LCFTs is significantly higher compared to short fiber based CFRTs. Additionally, LCFTs are easy to process and cost‐effective, showing great potential for replacing metals. The direct consolidation of LCFT (LCFT‐D) was initially developed at the Fraunhofer Institute in Germany using glass fibers. However, LCFT‐D technology for carbon fiber is still emerging, despite several decades of development.^[^ ^63^ ^]^ The LCFT‐D process involves semifinished pellets which are usually manufactured using injection or compression molding. The impregnation of the fiber and chopping of the fiber within the required length was driven by the potential savings in shipping and handling costs for the pellets by the adoption of a direct LCFT inline compounding facilities in the 1990s. This direct inline consolidation process combines injection or compression molding and in‐line compounding of fibers within the matrix, and the charge or the extrudate of the extruder is transferred onto a compression mold or the pellets directly fed to an injection mold to create the desired finished form.^[^ ^50^ ^]^ The fiber length of LCFT initial pellets is critical for enhancing mechanical and thermal properties in CFRTs such as the combination of carbon fiber and acrylonitrile‐butadiene‐styrene (ABS). Although there are merits to using a higher length of fiber in the LCFT pellets, the mechanical and thermal properties are usually function of the distribution of the fiber length and aspect ratio of the fibers in the composites, and have a less bearing on the mean length of the initial pallet as the length degenerates because of the shear forces in the extrusion and compression molding for injection molding process.^[^ ^61^ ^]^

##### Chopped Carbon Fiber Tape

Historically, randomly oriented discontinuous fiber systems were extensively utilized for compression molding of CFRPs. Their superiority lies in the complex shape design and flow of resin around the fibers. In automotive industry, the less energy‐intensive chopped carbon‐fiber‐tape‐reinforced thermoplastics (CTTs) with the randomly oriented discontinuous fiber systems has been widely applied in critical automobile components as they can reduce overall energy consumption and environmental impacts.^[^ ^64^ ^]^

**Figure 2:**
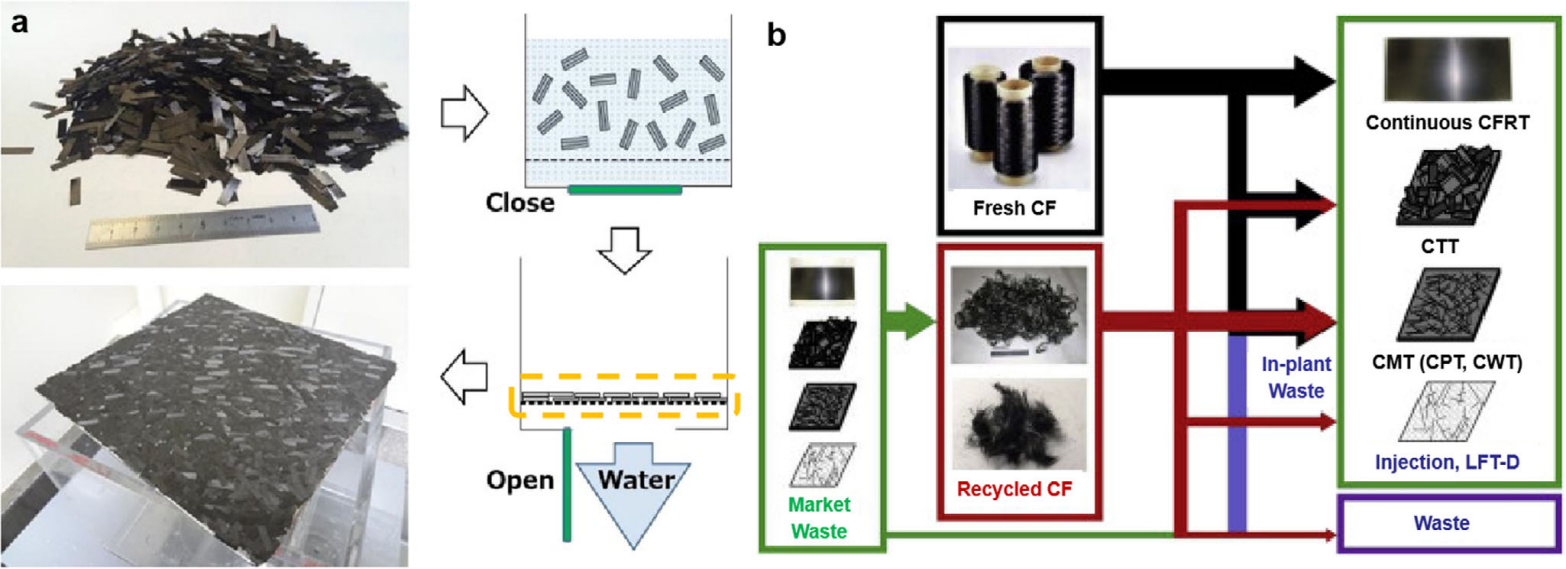
a) Representation of papermaking process for CTT composites. Adapted with permission.^[^ ^64^ ^]^ Copyright 2018, Elsevier. b) A schematic of producing CTT, carbon fiber mat reinforced thermoplastics (CMT), carbon fiber paper reinforced thermoplastics (CPT), and carbon fiber card web reinforced thermoplastics (CWT) from market waste and fresh CF. Adapted with permission.^[^ ^63^ ^]^ Copyright 2016, Elsevier.

The use of CTTs addresses a major shortcoming of CFRPs in terms of formability for complex shape components. The mechanical properties of the discontinuous carbon fibers are usually substandard or inadequate for structural components. With CTTs, the linearity or alignment of carbon fibers in producing the CFRT components with sufficient fiber aspect ratio can lead to high performance, affordability, formability, and recyclability of the CFRT components. CTTs particularly can be made with resin‐impregnated, unidirectional carbon fiber tapes with thickness less than 1/3 of the conventional tapes, which can lead to a higher performance in mechanical loading but with a smaller overall scatter. With a narrow cross‐section, the tape can impregnate resin 10 times faster than conventional methods due to its narrow cross‐section. Furthermore, since the tape is smaller, a papermaking technique can be used to disperse the resin within the carbon fiber preform (**Figure** **2**a).^[^ ^63^ ^]^ CTTs can also be obtained from chopping the compression‐molded waste from industrial CFRP products, such as the Gulfstream G650 elevator and rudder (Figure 2b). The CTTs obtained from these processes are shaped using a mold designed for thermoplastic‐based composites.^[^^]^ The major advantage of CTTs over traditional short fiber compounds is the ability to maintain a greater fiber length, allowing the compression‐molded composites to achieve superior mechanical properties because of higher degree of fiber alignment.^[^^]^ For CFRTs, the interlaminar shear strength and tensile strength are critical during failure and damage propagation. By increasing the fiber length, mechanical strength typically increases, but also scatter in strength values increases because of the lack of structural regularity as the fiber length increases.^[^ ^64^ ^]^

Overall, short fiber‐based composites either produced from virgin material or recycled material are capable of filling in the niche area where the long fiber compounds are unable or difficult to form because of their fiber length. A study on chopped carbon‐fiber‐tape‐reinforced PEEK composite was carried out to evaluate its molding process and mechanical properties, and the results showed that the tensile properties of this composite could be comparable to those of a carbon‐fiber‐reinforced epoxy composite, confirming its application value.^[^ ^65^ ^]^

### 3.3 Surface Treatment of Carbon Fiber

The interfacial property between carbon fiber and thermoplastic has a profound impact on the mechanical properties of CFRTs, but due to the different polarities of carbon fiber and thermoplastic, their interfacial strength is often low.^[^ ^66^ ^]^ Several processes have been applied to enhance the interfacial strength between carbon fiber and thermoplastic, such as physical adhesion, chemical bonding, and mechanical interlocking. These processes follow the principle of surface energy alteration, functionalization of the fiber surface, and ability to create rough fiber surface, respectively.^[^ ^67^, ^68^ ^]^ A high surface energy within the fiber compared to polymer ultimately results in adequate wetting and increase in contact surface area at the interface and hence dictates the optimal consolidation of the carbon fiber and the matrix within the composite structure. One big challenge in preparing an appropriate surface treatment is the high melt viscosity of thermoplastic, which often hinder the application of chemical interactions.

At a molecular level, the physicochemical interactions determine the interfacial adhesion.^[^ ^69^, ^70^ ^]^ The presence and preservation of van der Waals and hydrogen bonding between carbon fiber and thermoplastic matrix during consolidation are essential to retain a high range of mechanical properties. As a general principle, the interfacial adhesion energy requires to be greater than the cohesion energy offered by the thermoplastic matrix. The fundamental challenge with carbon fiber is the inertness of its surface due to a high temperature processing step during the fiber manufacturing. The low adsorption tendency of carbon fibers and the lack of physical defects on their surface lead to difficulties in adhesion to the thermoplastic matrix.

Carbon fiber manufacturers generally believe that general epoxy sizing or fiber coating is sufficient, leading to insufficient attention to carbon fiber surface treatment. However, with the increasing diversity in the applications of CFRTs and the need to cater to a larger range of applications at a lower carbon fiber cost have ultimately driven the industry to engage in novel surface treatments for carbon fiber used in CFRTs. Carbon fiber surface treatments can be classified as wet chemical method and dry mechanical interlocking method. For wet chemical method, chemical coating, grafting, sizing, and anodic oxidation techniques are widely used. Dry mechanical interlocking method employs a plasma, ozone, or gamma incidence to gain the desired fiber surface roughness. Often these distinct methods of enhancing interfacial adhesion work synergistically.^[^ ^69^ ^]^ A balance of physical adsorption and chemical interactions dictates the effectiveness of surface treatments. Nonetheless, to conduct a fiber surface treatment, the existing thermoset coating on the carbon fiber needs to be removed. Upon successful interface engineering, a phase additional to the fiber and matrix is manifested, which often can be characterized as a distinct phase that assists in the fiber/matrix load transfer through interfacial shear stress transfer.^[^ ^71^ ^]^

## 4 Processing and Postprocessing Methods of CFRTs

We discuss traditional and automated processing methods for CFRTs, including techniques to produce near net shape parts using automated methods and traditional methods of impregnating carbon fiber with a thermoplastic polymer.

### 4.1 Automatic Fiber and Tape Placement

Automated fiber placement (AFP) and automated tape placement (ATP) are advanced manufacturing methods for CFRTs, which are designed to automate the layer‐by‐layer lay‐up process. Both AFP and ATP enable high‐throughput production of advanced composite laminates using unidirectional preimpregnated (prepreg) tows or tapes,^[^ ^72^ ^]^ and a high potential of savings in terms of process speed and cost. These methods allow for customized lay‐ups and near net‐shaped blanks, which are aimed at reducing weight and waste but still with high strength, making them suitable for primary load‐bearing parts.^[^ ^73^ ^]^

**Figure 3:**
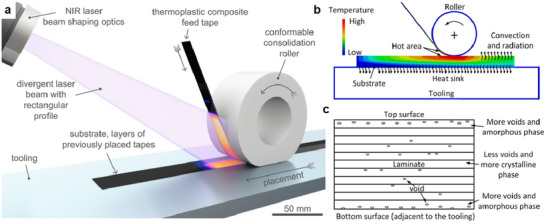
a) Illustration of LATP using laser as a heat source, b) temperature distribution of the incoming tape and substrate during ATP, and c) matrix morphology after ATP. Reproduced under the terms of the CC‐BY Creative Commons Attribution 4.0 International license (https://creativecommons.org/licenses/by/4.0).^[^ ^74^ ^]^ Copyright 2024, The Authors, published by Elsevier.

In these processes, a unidirectional tape is robotically laid on the top of the previously placed layers. The tape and substrate surfaces are heated by a heat source such as an infrared, heat gun, or laser while the tape or laminate is in motion (**Figure** **3**a). The tape surface is heated near the nip point, where it meets the substrate to enable intermolecular diffusion of the thermoplastic matrix (Figure 3b). By pressing the two melted surfaces together with a compacting roller, the layers are bonded together to reduce porosity and achieve optimal adhesion.

The ATP/AFP process usually requires ultrasonic tacking that involves applying mechanical vibrations with high frequencies (20–50 kHz) and low amplitudes (10–250 µm), combined with a static welding force to bond layers together. Continuous ultrasonic tacking is used for 2D tape laying and three‐dimensional (3D) fiber placement of CFRTs. Technically, achieving high laydown speeds with high layup accuracy is a challenge. Common laydown speed with ultrasonic tacking is ≈0.5 m s^−1^ for carbon/PEKK lay‐ups and up to 2 m s^−1^ for low‐end UD tapes. Gantry robots monitor the lay‐up accuracy to a resolution of ± 0.30 mm to adhere to aerospace specifications.^[^ ^75^ ^]^

The finish quality after ATP/AFP process is a delicate balance between laying speed, porosity, crystallinity, and tape thickness. Porosity in consolidated parts is resultant of ATP/AFP rate, part geometry, and tape quality, which is in turn an effect of deviations in thickness because of the resin and fiber distribution at the microstructural level (Figure 3c). Fiber placement in flat panels can be altered to achieve increased consolidation, which is driven by the processing temperature and compaction pressure, removing the intraply porosity inherent to tapes.

Crystallinity is another key challenge, which is controlled by the cooling rate. To reduce residual stress buildup and dimensional instabilities such as shrinkage or warpage, recrystallization is designed to be completed entirely within the production cycle. Furthermore, nonhomogeneous cooling of part surface leads to shrinkage as well, which is remediated by adjusting tool temperature.^[^ ^76^ ^]^

Laser‐assisted tape placement (LATP) is a variant of ATP that uses UD tape based CFRTs with consolidation achieved out‐of‐autoclave along with automation capabilities (Figure 3). In LATP process, the temperature, pressure, and speed are adjustable to achieve in situ consolidation. It has been extensively documented in the literature that process parameters influence mechanical performance and bonding quality. An understanding of laser irradiation and reflection near the nip point is crucial for successfully designing in situ consolidation. As the composite tape absorbs and reflects laser light differently at different angles, LATP is often difficult to control. For proper consolidation and adhesion between layers, the desired nip point temperature must be maintained despite material, geometry, and process parameters.

### 4.2 Postconsolidation and in situ Consolidation

CFRPs have been effective in the production of flight‐critical structures with minimal level of flaws such as surface and section porosity, which affect predictable and reliable performances. Post consolidation in autoclave is a reliable method of obtaining a porosity level of <1 vol.%, but this involves high capital and operational expenditure. To reduce such high costs, out‐of‐autoclave (OOA) consolidation technology was developed. Ho et al. prepared a PEKK UD tape‐based laminate using ATP at 600 mm s^−1^ and subsequent post consolidation with vacuum bag in an oven.^[^ ^53^ ^]^ Without post consolidation, the laminate exhibited intraply porosity, confirming the requirement of post consolidation. In addition to reducing cycle time, OOA consolidation also reduces energy consumption. One prime example of OOA technology is CFRT ribs for Airbus that were designed as replacements for aluminum alloy counterparts. In situ consolidation (ISC) of CFRTs during automated lay‐up is similar to forming, offering full consolidation of laminates with a low level of porosity and desired mechanical properties in a single step. To accommodate ISC, often the ATP speed is reduced to obtain the desirable level of consolidation. For instance, Ho et al. reduced the ATP speed to 200 mm s^−1^ to yield minimal porosity for a LMPAEK UD tape‐based laminate.^[^ ^53^ ^]^

Balance of speed and quality is challenging in AFP, with conventional wisdom stating that a faster process yields a lower‐quality yet economical part. Autoclaving allows fast AFP speeds and still meets the low porosity requirement. ISC AFP is slower, but would be practical for fuselages, not wings because of thickness variations. A thick wing structure prevents ISC, as opposed to a thin fuselage. ISC today can be competitive at current speeds (60–100 mm ^−1^s) without the need for secondary steps. ISC is still developing in the industry, with ongoing research focusing on production speed and process robustness.^[^ ^55^ ^]^ Achieving near‐net shape as well as reducing waste are not a function of machine speed, albeit requires knowledge of issues that can slow production, for example, fiber orientations, building‐up plies, and stopping and restarting.

In summary, ATL and AFP are fast automated techniques used to create simple to mildly‐curved laminates such as aircraft components such as wing skins, frames, stringers, wing boxes, and fuselage structures. Prior to full commercialization, several challenges still need to be overcome, including achieving an appropriate level of crystallinity when using semicrystalline thermoplastics and reducing residual stresses that can cause delamination. In addition, the higher porosity level inherent to AFP also prevents it from achieving greater acceptance in the aerospace industry.^[^ ^72^, ^77^ ^]^

### 4.3 Thermoplastic Tape Winding

**Figure 4:**
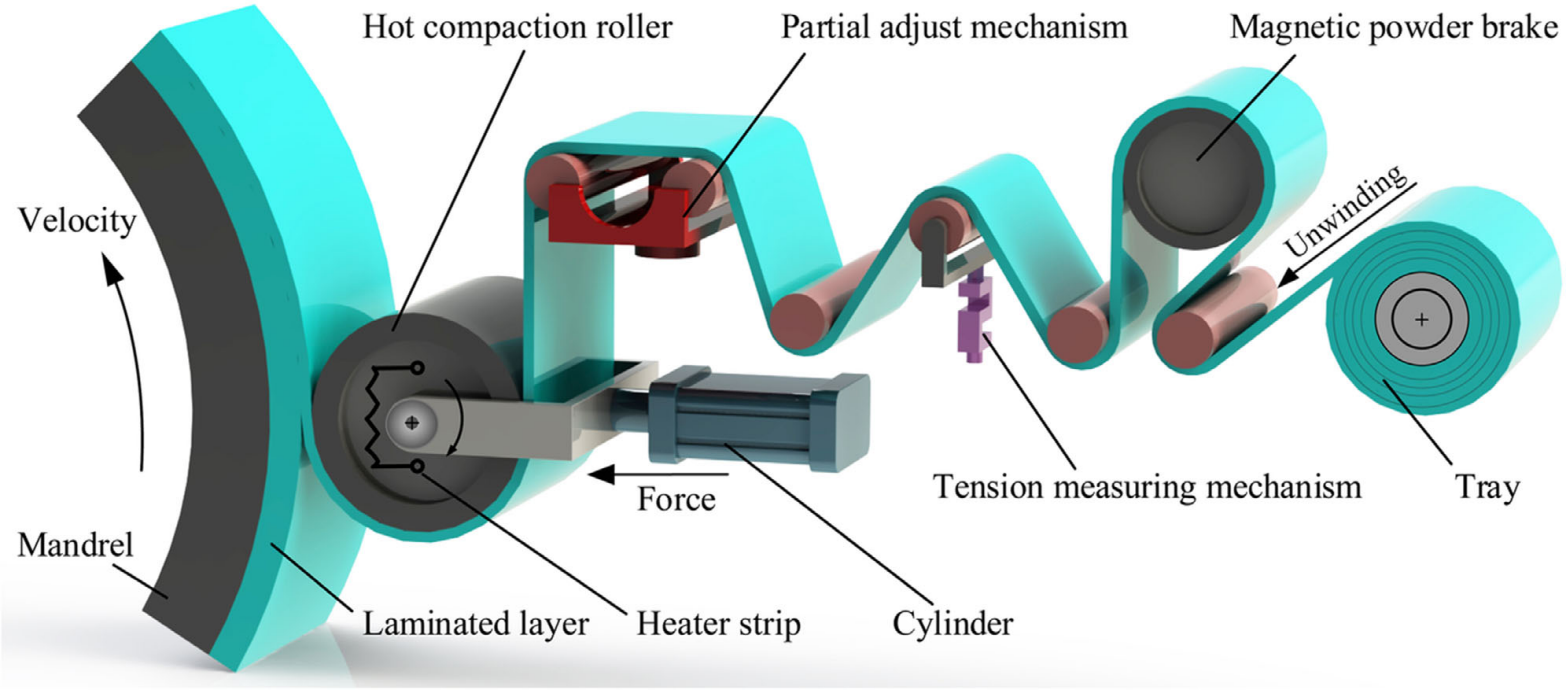
Schematic of TTW process. Adapted with permission.^[^ ^79^ ^]^ Copyright 2018, Springer Nature.

Thermoplastic tape winding (TTW) is a highly automated method for manufacturing tanks, tubes and pipes in water supply and oil and gas industry. Tape winding is a variant of filament winding and a typical tape winding process is shown in **Figure** **4**. Generally, this process uses a cylindrical mandrel/tool to produce circular components. Heating sources to achieve the in situ consolidation are similar to those used in AFP and ATP. Flat, circular, as well as elliptical laminate sections can be made using TTW.^[^ ^72^, ^78^ ^]^

In TTW, the complete in situ consolidation is achieved during the winding stage, with no post processing or secondary thermal treatments necessary. Hence, the temperature at the nip point is a critical processing parameter for TTW. Incoming tape fuses instantly with the underlying substrate, allowing for geometries not feasible with traditional filament winding. Furthermore, the instant consolidation allows inclusion of additional plies at strategic locations to achieve local stiffening. Importantly, TTW eliminates fiber slippage and provides optimal stability for winding paths in nongeodesic trajectories. TTW enables the production of complex shapes and product trials easier over traditional, thermoset filament winding counterparts.

Production speed and quality are essential for the CFRT components made by thermoplastic tape winding. Bonding quality is influenced by rotation and tape tension, but not many studies have examined the effects of tape tension and winding kinematics on bonding quality.^[^ ^78^ ^]^ Key process parameters, such as temperature, applied consolidation force, and winding speed, determine the interlaminar bond strength. Bonding degree is directly influenced by the degree of initial consolidation of the tape, which is affected by temperature, humidity, and tape storage time. Therefore, careful attention to the storage process of prepreg tape is necessary to reduce the initial cure degree.^[^ ^79^, ^80^, ^81^ ^]^

### 4.4 Stamp Forming

**Figure 5:**
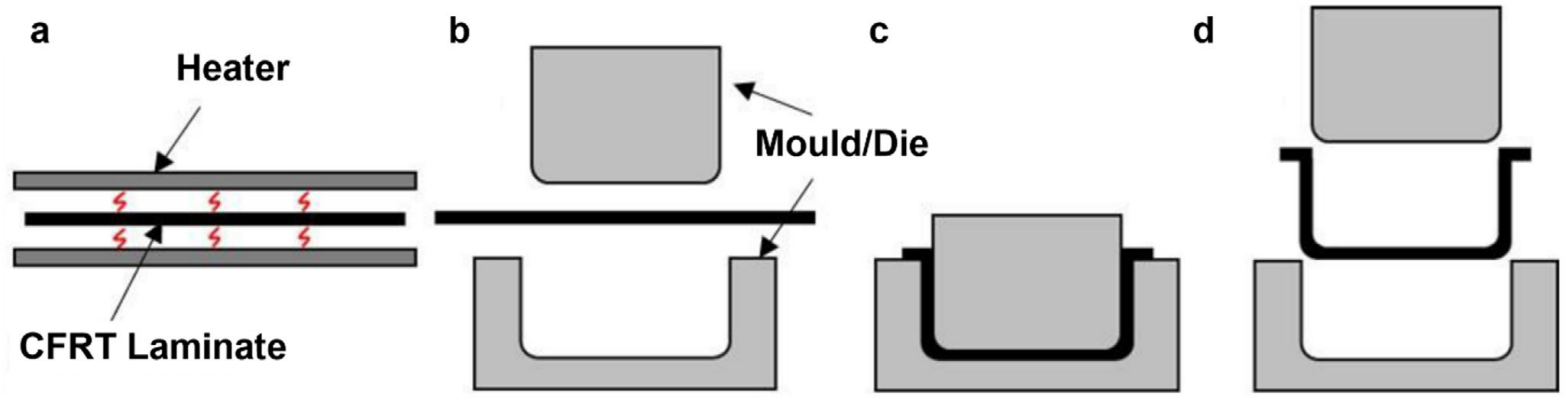
Schematic of a stamp forming process: a) heating, b) transfer, c) forming, and d) removal. Reproduced under the terms of the CC‐BY Creative Commons Attribution 4.0 International license (https://creativecommons.org/licenses/by/4.0).^[^ ^83^ ^]^ Copyright 2022, The Authors, published by Elsevier on behalf of KeAi Communications.

In stamp forming, flat CFRT sheets are pressed and formed into components. A short cycle time makes this process highly appealing for large‐scale production in the automotive and aerospace industries, with the most common applications being straight and slender floor beams. However, stamp forming cannot produce certain geometries such as a double‐sided bottom flange component on a rib.^[^ ^82^, ^83^, ^84^, ^85^ ^]^ A typical stamp forming process is illustrated in **Figure** **5**. After tape laying, a laminate is consolidated either in situ or post consolidation, as introduced previously. Then, the laminate is pretrimmed to a suitable geometry for subsequent forming to reduce fiber wrinkling.^[^ ^86^ ^]^ The laminate is then heated to a flowable status at processing temperatures over the *T*_m_ for semicrystalline and about *T*_g_ for amorphous polymers using infrared heating. Springs or frames are used to avoid contact between laminate and heating elements. A laminate is swiftly moved to a press station for forming 3D. During cooling, consolidation pressure is held until the component is released from the tool. Since the tooling temperature is kept between the *T*_g_ and *T*_m_, the laminate is effectively quenched. Cycle time for stamp forming is typically in the range of a few min.^[^ ^82^ ^]^

Porosity, interlaminar bond strength, and crystallinity of semicrystalline polymers are all determined by the consolidation quality of a stamp‐formed component. These properties are an effect of their thermal and pressure histories. Consolidation degree increases during preconsolidation; however, deconsolidation can occur at high temperatures in absence of external pressure during heating, resulting in increased porosity and ply delamination, requiring corrective re‐consolidation. AFP blanks undergo decrease in thickness and porosity upon application of stamp forming. Predominantly, porosity is confined to interfaces between plies, rather than within the plies.^[^ ^82^, ^87^, ^88^, ^89^ ^]^ Friction between plies and out‐of‐plane bending are induced during stamp forming, resulting in fiber wrinkling, waviness, breakage, and air voids.^[^ ^90^, ^91^ ^]^ Higher forming rates limit inter‐ply slip, resulting in increased surface fiber buckling. A rapid approach followed by slower forming and consolidation period is recommended. At higher forming rates with warmer tools materials conform fully, while cool molds cause matrix solidification before complete part drape, limiting inter‐ply slip.^[^ ^90^, ^92^ ^]^

Maintaining consistent pressure across the entire product surface is challenging. Cooling during solidification dictates the morphology and mechanical properties of semicrystalline CFRTs. Faster cooling reduces crystallinity and crystallite sizes and increases interlaminar fracture toughness and decreases transverse elastic modulus, but importantly creates temperature gradients through cross section, causing internal stresses because of heterogeneous and anisotropic shrinkage. Semicrystalline CFRTs also experience nonisothermal solidification due to crystallinity gradients, which can arise from stacking sequence and resultant mismatches in stiffness/thermal expansion amongst plies, leading to internal stresses and reduced strength, cracks, or delamination, which can result in premature failure. Posttreatments like annealing can relax these stresses but are often expensive and time‐consuming.

### 4.5 Compression Molding

#### 4.5.1 Conventional Compression Molding

Compression molding is commonly used for producing discontinuous CFRTs such as LCFT composites, which applies high pressure and temperature to squeeze out preheated LCFT pellets and fill a mold to ensure consolidation.^[^ ^24^ ^]^ Processing CFRTs containing longer fibers can be realized by compression molding to obtain greater mechanical properties than traditional short‐fiber compounds. Fiber length and orientation are critical as load bearing reinforcements, and fibers are prone to breakage because of shearing and fiber‐fiber interaction and entanglement. To retain the mean fiber length, a low‐shear application during the mold filling is required.^[^ ^50^ ^]^

Fiber orientation within compression molding is a function of the processing conditions, including charge deposition and flow. During molding, partially covered molds may cause a preferential alignment of fibers in the flow direction. The establishment of a relationship between the molding conditions, fiber orientation, and mechanical property development is crucial for its efficient use in intended applications.^[^ ^93^ ^]^

Often, extrusion/compression molding is used to reprocess recovered carbon fibers as CFRTs. For example, the recyclate from retired Gulfstream G650 elevator and rudder was developed as recycled flakes and applied to produce an access door panel. This door panel featured advanced structures such as stiffening ribs and bosses.

#### 4.5.2 Continuous Compression Molding

**Figure 6:**
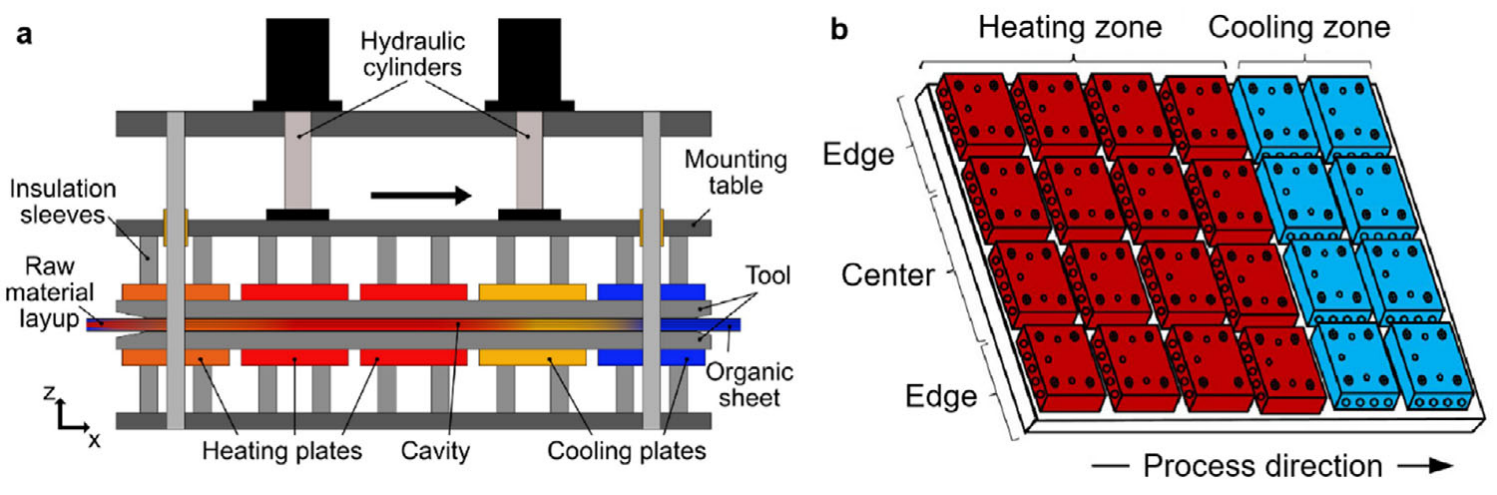
a) Schematic of CCM showing the key zones and components. Reproduced under the terms of the CC‐BY Creative Commons Attribution 4.0 International license (https://creativecommons.org/licenses/by/4.0).^[^ ^98^ ^]^ Copyright 2021, Neue Materialien Fürth GmbH, published by Taylor & Francis. b) Illustration of modular heating and cooling unit of the CCM machine. Adapted with permission.^[^ ^99^ ^]^ Copyright 2016, Sage Journals.

Continuous compression molding (CCM) is a technique for continuously manufacturing consolidated preforms or components starting from a CFRT sheet. Producing dimensionally accurate, high‐quality components in large quantities is achievable with CCM. Compared to other CFRT manufacturing processes such as hot forming or stamp forming, CCM offers several advantages, including a high production rate and the ability to produce large components. However, CCM does have some limitations, such as high initial tooling costs and limited flexibility for small batch production.^[^ ^94^ ^]^ **Figure** **6**a shows the positioning of the CFRT sheet on a linear steel support, which is guided through an open double mold. The sheet is preheated above its *T*_m_, pressed, and cooled under pressure (Figure 6b), and then a feeder pulls the formed part out of the mold. After exiting the tool, the laminate is fully impregnated, consolidated, and solidified.^[^ ^95^ ^]^ The maximum thickness achievable in CCM is limited by the process speed, and the production speed for shaped profiles is slower than flat panels. A key feature of the CCM process is its ability to modify part lay‐up by adding or reducing layers without interrupting the process, simply by replacing the feedstock sheet layer. Prepreg including film stacked and powder prepregs made from woven or nonwoven fabrics or randomly oriented textiles (such as glass, carbon, aramid, or natural fibers) are as reinforcement for CCM today. There is a wide range of thermoplastic matrices that can be used, including PP, PA, and PEEK depending on the application and desired long‐term service temperature. With CCM, laminate thickness ranging from 0.5 to 6 mm and up to 40 mm for sandwich structures are possible.^[^ ^96^ ^]^

Organosheets are formed using static press, (semi‐) continuous CCM. Melt viscosity of the thermoplastic defines the impregnation time, which essentially controls the volume of output. Generally, higher temperatures (lower viscosity) and higher pressures bring about the reduced impregnation time. The impregnation process for woven preforms involves macro‐impregnation (penetration around fiber bundles) and micro‐impregnation (infiltration of fiber bundles) phases. The permeability of a fiber bundle is up to 200 times higher for polymer flow parallel to the fibers compared to orthogonal flow. In‐plane polymer flow can accelerate impregnation. However, permeability changes occur during the impregnation process.^[^ ^97^ ^]^ After the polymer melts, external pressure compresses the fibers, and the matrix begins to penetrate into the fibers, reducing textile compaction. Impregnation is completed once the fiber bed is saturated, but fibers may not be evenly distributed across the thickness of the laminate, leading to fiber bed relaxation. 2–4 MPa is the optimal pressure range for thermoplastic impregnation. Achieving homogeneous pressure distribution is challenging during CCM because of the configuration of the equipment, but also a result of lateral and through‐thickness polymer flow.

The primary process parameters for CCM include feeding speed, temperature, pressure and dwell time.^[^ ^95^ ^]^ These possess an effect on spots and porosity, fiber washing and wrinkling, thickness distribution, and delamination.^[^ ^95^ ^]^ The quality can be optimized by adjusting the processing parameter, and both molding pressure and temperature are directly related to consolidation quality. Typically, pressures of 2.5 MPa are applied, with feed rates ranging from 30 to 80 m h^−1^. Preheated tools (below preheating temperature but above room temperature) reduce internal stresses caused by rapid cooling and prevent amorphization. The cooling rate can also be controlled by adjusting the temperature along the press and the speed at which the part moves through the press.

Airbus and Boeing have used CCM for many years and now produce shaped consolidated laminates. CCMs are great for cost, but the cross‐sections must remain constant. CCM can also be used to produce continuous profiles like L‐, U‐, and V‐shapes, as well as flat, “U,” “I,” “II,” and closed hollow sections. CCM is also used to manufacture parts with symmetric and asymmetric profiles, such as T‐ and J‐profiles, and structural components in aerospace. CCM products exhibit constant thickness and lay‐up with theoretically unlimited length as the sheet can be spliced. The versatility of CCM lies in producing a range of cost‐effective parts including straight or modified parts ranging to constant radius curved parts. In process variations in thickness and curvature radius are currently not possible with CCM, but are currently under development.

#### 4.5.3 Prepreg Platelet Molded Composites

A prepreg platelet molded composite (PPMC) is produced by compression molding UD slit tapes cut into a defined width and length. Platelets are composed of identically aligned and lengthened fibers, as well as having a fixed volume fraction. In addition to possessing a similar thickness to the original tape, they consist of 50–60% fibers, which is the ideal range for optimizing mechanical performance while retaining the processability needed for creating complex geometries. During processing, uncontrolled material deposition and anisotropic flow in the mold lead to stochastic meso‐structures in PPMCs, which result in inconsistency in mechanical properties.^[^ ^100^ ^]^ The fibers remain aggregated and locally collimated when a platelet is molded. The platelets in each bundle are curved around adjacent platelets resulting in crimped overlaps, producing a nonplanar, semilaminated morphology.

Orientation of a platelet in an aforementioned stochastic meso‐structure dictates its mechanical properties and is often driven by charge deposition and flow during processing. A global, planar random distribution of platelet orientations is obtained when charge fills the mold, while partially covered molds lead to preferential alignment in the flow direction. If a stochastic PPMC is to be used effectively, it is imperative that a relationship is established between the molding conditions, the platelet orientation state, and mechanical properties distribution.

PPMC analysis often involves a representative volume element approach with unidirectionally stacked platelets to model the platelets and their interfaces.^[^ ^101^, ^102^ ^]^ Progressive failure analysis is conducted using continuum damage mechanics and studying cohesive behavior at the interface to calculate a critical platelet aspect (*l*/*d*) ratio where failure changes from delamination to fiber breakage. Often, a 2D model is used to predict the apparent PPMC strength by determining the effects of meso‐structure and platelet failure.^[^ ^65^ ^]^

The modeling and analysis of PPMCs are challenging, and often involve complex frameworks to predict properties. Past studies have attempted to treat stochasticity of PPMC materials using different abstraction levels, such as idealizing the meso‐structure. A stochastic laminate analogy can be used to estimate for PPMC modulus variability as modeling alone is not capable to estimate platelet‐to‐platelet stress transfer and to effectively address platelet thickness and length effects.^[^ ^103^ ^]^ Representative volume elements with embedded element for discontinuous fiber composites is another common approach, which can lead to accurate stress transfer prediction.^[^ ^104^ ^]^ Developing a high‐fidelity computational analysis based on a discrete damage model could provide a new level of sophistication.^[^ ^101^ ^]^

### 4.6 Injection Overmolding

**Figure 7:**
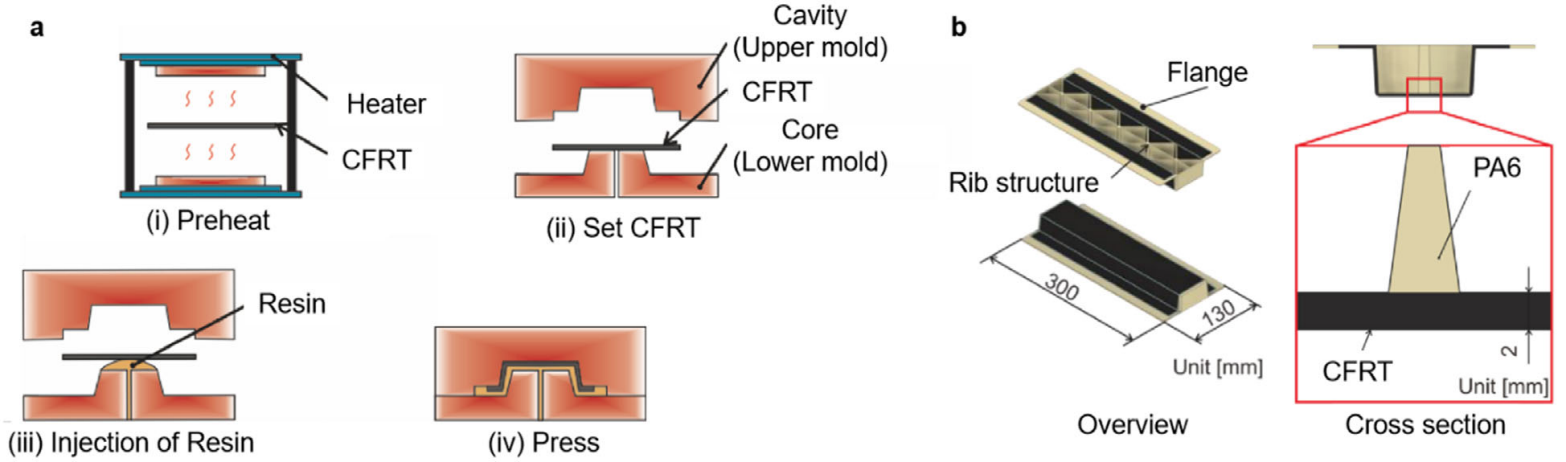
a) Illustration of CFRT injection overmolding process: i) Preheat, ii) Set CFRT, iii) Injection of resin, and iv) Press. b) Overview and cross section of shell‐shaped CFRT structure. Reproduced under the terms of the CC‐BY Creative Commons Attribution 4.0 International license (https://creativecommons.org/licenses/by/4.0).^[^ ^108^ ^]^ Copyright 2024, The Authors, published by MDPI.

In overmolding, a plastic or composite part is sequentially covered with another plastic or composite part from the same chemical family.^[^ ^105^ ^]^ Overmolding of organosheet is widely established that integrates tape with laminate. Thermoplastic composite overmolding (TCO) involves the combination of thermoforming and injection molding, to enable the manufacture composite structures that contain continuous and short fiber reinforcements in a very short time frame. An organosheet is heated and thermoformed during the closure of the mold plates, and the heated laminate is then overmolded with a short fiber‐reinforced material (**Figure** **7**a).

Overmolded structures feature geometric complexity due to the short fiber‐reinforced architecture, which can be used for stiffening, impact absorption and functional purposes. In addition, thermoplastics can be processed very quickly, resulting in very low cycle time for manufacturing such components.^[^ ^106^ ^]^ The bond strength between the CFRT sheet and the injected polymer is the limiting factor in this process, dependent strongly on thermal history. The interface temperature, therefore, is as important as other parameters such as pressure and viscosity.^[^ ^107^ ^]^

The lightweight potential of continuous fiber reinforced plastics is higher for structurally relevant parts. Figure 7b shows an example of thermoforming and injection molding combined with CFRTs, with a relatively high degree of design freedom. Shell‐shaped structures made of continuous fiber CFRT can therefore be reinforced with ribs and overmolded to add additional functional elements.

The design of the part and the process plays an important role because of the flow path and fill time during overmolding.^[^ ^109^ ^]^ The sheet temperature distribution is inhomogeneous because of the contact conditions and inhomogeneous temperatures. Recent successes in hybrid lightweight structures made by overmolding have been driven by new requirements for mass reduction and cost efficiency in the development of components, such as those for mobility applications.^[^ ^110^, ^111^ ^]^

Injection overmolding CF‐PPS ribbed plates were used to understand how processing parameters affect yarn deformation, fiber orientation, void content, and displacement of an organosheet.^[^ ^112^ ^]^ Pressure profile and clamp force are the most important factors affecting yarn deformation. The quantity of matrix material displaced from pressed organosheets without overmolded short‐fiber material increases with clamp force and preheating temperature.^[^ ^106^ ^]^

Injection overmolding parameters are often optimized for interfacial strength in hybrid composites. A hybrid PA‐based CFRT structure was built by Fiorotto and Lucchetta.^[^ ^113^ ^]^ Preheating an organosheet at temperatures higher than the *T*_m_ of PA can lead to a significant increase in bonding strength compared to other processing parameters. Tanaka et al. showed that mold temperature and preheating lead to an increase in penetrated depth and the increased tensile strength of hybrid composites.^[^ ^108^ ^]^ Using preheat organosheet prior to injection overmolding increases hybrid composite bonding strength.

If molten polymers with high temperatures were injected on the surface of the organosheet, they generally encountered intimate contact and self‐diffusion. Through MD simulations, Jiang et al. examined how melting temperature and injection pressure affect hybrid composite interfacial characteristics.^[^ ^114^ ^]^ Diffusion coefficients and bonding energies increased with melting temperatures and injection pressures. Simulating hybrid composites at the atomic or molecular level with MD simulation is a practical way to characterize their interfacial behavior.^[^ ^115^ ^]^ The physical testing of overmolded structures is conducted through tensile rib pull‐off to evaluate bonding at an overmolded interface. Rib geometry and sampling location determine process‐induced features, cohesive failure occurs at higher loads, and adhesive failure at the interface indicates a weaker joint.^[^ ^116^ ^]^

### 4.7 3D Printing

3D printing, as a common additive manufacturing method, has received increasing attention over the past years, which presents high design flexibility without any need on mold, and cost saving.^[^ ^117^, ^118^ ^]^ 3D printing can be utilized in the manufacturing of prototypes, jigs, fixtures, molds, and parts.^[^ ^119^ ^]^ Due to the tailored process, less material will be wasted. 3D printing includes several technologies, such as selective laser sintering (SLS), fused deposition modelling (FDM), and stereolithography (SLA). FDM is the most widely used 3D printing method.^[^ ^120^ ^]^ Both continuous and discontinuous fibers could be used in 3D printing.^[^ ^121^ ^]^ Compared to continuous fiber, discontinuous fiber reinforced thermoplastics have been intensively investigated and their 3D printing techniques are relatively mature.^[^ ^118^ ^]^ Continuous fiber reinforced thermoplastics could be printed using in situ fusion technology or *ex‐situ* technology.^[^ ^118^ ^]^ In situ fusion provides a variable fiber volume content but suffers from poor adhesion at interface due to short dwell time.

The quality of 3D printed parts is decided by many factors, such as fiber content, cracks, porosity, and adhesion between printed layers, and it significantly influences the interlaminar strength and tensile properties. A higher fiber content could reduce the interface contact between fiber and matrix, leading to cracks.^[^ ^122^ ^]^ Thus, the fiber content should be carefully controlled. According to previous work, when the CF content was above 20%, the printed samples usually showed poor mechanical properties.^[^ ^123^, ^124^ ^]^ In addition, a higher porosity could result in interface failure and thus reduced mechanical properties.^[^ ^122^ ^]^ The adhesion between printed layers play an important role in the interlaminar strength of the printed parts.

SLS is the another widely investigated 3D printing technology, but it uses composite powder. Compared to FDM, the fibers used in SLS are in nano or micro range, which limits the application of the printed products under high load. Meanwhile, due to the anisotropy of carbon fibers, the powder spreading stage needs more careful control.^[^ ^118^ ^]^

## 5 Welding of CFRT Parts

Aerostructures require advanced joining technologies to maintain structural integrity.^[^ ^125^ ^]^ One of the main joining techniques for CFRTs is thermoplastic welding because the thermoplastic matrix is weldable. The thermoplastic welding is accomplished by applying heat and pressure locally to the material. This thermoplastic welding allows very short processing time (seconds to min) and is fastener‐free, which has the potential to assemble large structures such as fuselages efficiently.^[^ ^126^, ^127^ ^]^

During the welding process, heating causes the thermoplastics to reach a viscous state at their interfaces to achieve physical entanglement of their polymer chains, which then forms a join after cooling.^[^ ^128^ ^]^ Welded parts are of similar quality to autoclave‐consolidated and compression‐molded components.^[^ ^36^ ^]^ The outward appearance of a weld is seamless, as the polymer chains are amalgamated across an interface causing any weld lines to disappear at the joint surfaces, enabling transfer of loads through the welded area. Thermoplastic welding can also eliminate time and labor requirements for the fastening and the fasteners themselves that are usually made with Ti alloys.^[^ ^129^ ^]^ Various thermoplastic welding techniques have been developed in recent years. Among these, ultrasonic welding (UW), induction welding (IW), resistance welding (RW), and laser welding (LW) are most applicable for CFRTs.^[^ ^130^ ^]^

### 5.1 Ultrasonic Welding

**Figure 8:**
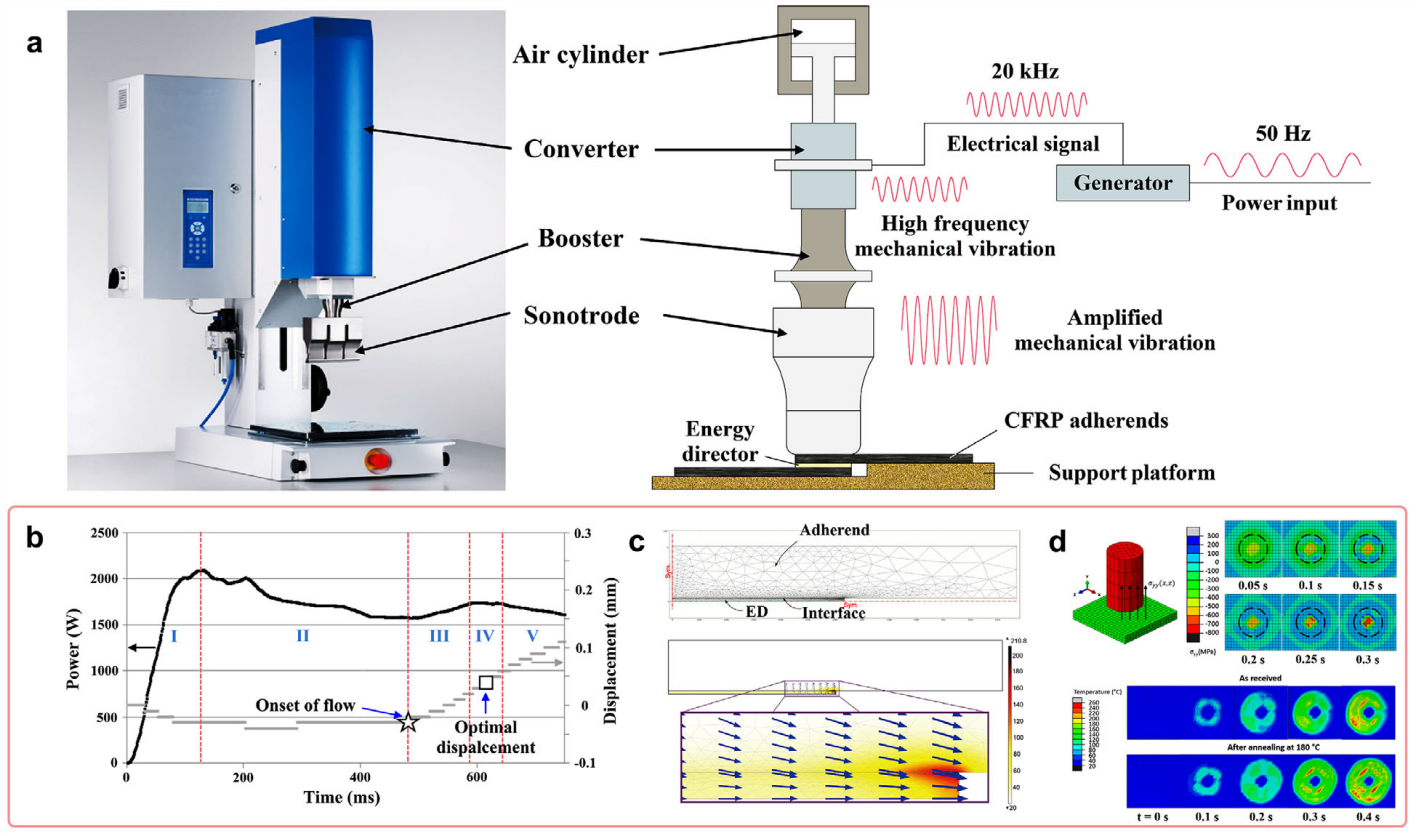
a) Schematic of UW equipment, (b) five stages of UW process, and c, d) simulated results of the heat generation at the welding interface. Adapted with permission.^[^ ^131^ ^]^ Copyright 2025, Elsevier.

The UW equipment is composed of an assembly press, nest or anvil, ultrasonic stack, vibration converter, amplitude booster, a sonotrode, and an ultrasonic generator (**Figure** **8**a).^[^ ^88^, ^132^, ^133^, ^134^, ^135^, ^136^ ^]^ The UW process can be divided into five stages: interfacial heat generation (i), interface partial melting (ii), melted resin extrusion (iii), interface balance state (iv), and interface edge over‐heating (v) (Figure 8b).^[^ ^137^ ^]^ The highest welding strength can be achieved in stage (iv), and the over‐heating in stage (v) will destroy the welding of the joints (Figure 8c,d).^[^ ^138^ ^]^

In the aerospace industry, UW is widely used to join CFRTs, and research has been conducted to determine the best parameters and process windows for high‐quality CFRT welding.^[^ ^133^, ^135^ ^]^ Kagan and Nichols reported that the lap joint shear strength of ultrasonically welded PPS/carbon fiber laminates can be up to 30 MPa.^[^ ^29^ ^]^ Although UW has been widely utilized to joint CFRT parts, it is still difficult to obtain seamless welds for large structures by continuous UW.^[^ ^139^ ^]^ Optimizing velocity and energy are key elements for achieving continuous UW with specific materials and laminate thicknesses.

### 5.2 Induction Welding

**Figure 9:**
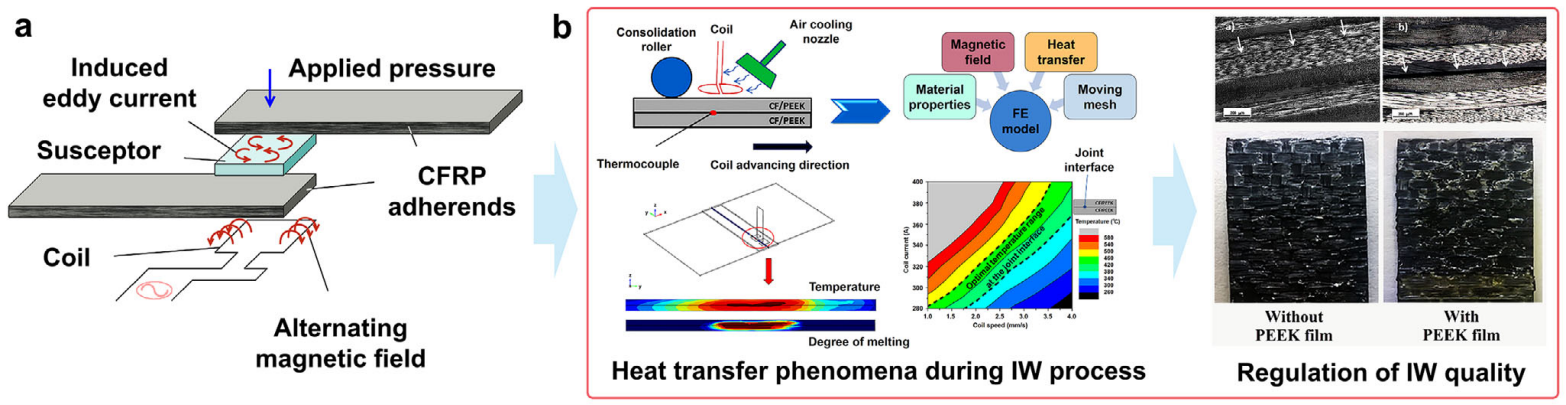
Schematic of a) IW process and b) enhanced welding quality of the CF/PEEK joints. Adapted with permission.^[^ ^131^ ^]^ Copyright 2025, Elsevier.

IW involves the use of an induction coil to generate a high‐frequency alternate magnetic field.^[^ ^140^ ^]^ A magnetic field creates eddy currents inside the laminate under the carbon fiber reinforcement, resulting in localized heating of the thermoplastic matrix. Then, the molten resin segments at the interface are entangled with each other under external pressure to form a weld (**Figure** **9**a).^[^ ^29^ ^]^ The heating generation modes of IW include Joule loss, dielectric heating and contact resistance heating. As a contrast, IW parameters, such as laser power, laser frequency, and welding pressure, have significant effects on the quality of CFRT parts made with IW. Welding speed and welding strength are directly related to the laser power and frequency of the laser, as well as the welding pressure.^[^ ^141^ ^]^ A major defect of IW compared to other methods of welding is its inability to regulate temperature along the joint thickness and to avoid excessive temperatures on surface immediately exposed to an induction coil and close to the edges.^[^ ^142^ ^]^ To improve the welding temperature uniformity and stability, Lionetto et al. applied a layer of thermoplastic films on the CFRT parts (Figure 9b), and developed a temperature distribution model of the CFRT parts during IW. Then, the processing data could be regulated to achieve the optimal processing window.

There are some issues, such as mechanical performance difference between metal conductor and thermoplastic matrix and poor heat generation of graphite or carbon fiber, that limit the further application of IW in aviation CFRT parts. Hence, the exploitation of new conductors with high heat generation performance and good compatibility with thermoplastic matrix has become a major development direction of IW for CFRTs.

### 5.3 Resistance Welding

As a joining technique for CFRT parts, RW is considered promising. RW processes use conductive elements, such as carbon fiber prepreg and metal mesh, to clamp together CFRT laminates at their interfaces.^[^ ^29^, ^132^, ^143^ ^]^ Then, the adjacent laminate surfaces are quickly heated to melting temperatures when the electrical current passes through the conductive element. A limited melting zone at the interface is created to achieve welding, which can also ensure the shape and dimensional stability of the components. As the welding time for this process ranges between 1 and 4 min, RW can be used on large structures.^[^ ^144^ ^]^ The welding current and heating time have a surprising influence on the efficiency of the interface heat generation and which, in turn, affects the welding strength of CFRT joints. In addition, proper welding pressure and conductive element are two key factors in achieving ideal RW CFRT joints.^[^ ^29^, ^132^, ^145^ ^]^

For RW, the interface temperature uniformity is particularly important to achieve high welding strength. In addition, an ideal conductive element should have good compatibility with the thermoplastic matrix and generate uniform heat at the interface. However, as conductive elements, the carbon fiber prepreg and metal mesh suffer from poor heat generation uniformity and unsatisfactory compatibility with thermoplastic matrix, respectively. Obviously, there is a trade‐off between compatibility between conductive element and thermoplastic matrix and interface temperature uniformity. To solve this issue, it is necessary to explore new conductive elements for RW of the CFRT parts.

### 5.4 Laser Welding

LW is also a promising technique for joining CFRT parts because it does not require conductive element and has fast welding rate and low mechanical stress.^[^ ^146^ ^]^ Laser transmission welding is the most widely investigated and applied technique for joining CFRT parts among various LW techniques.^[^ ^126^ ^]^ Laser transmission welding involves passing the laser radiation through the upper laser‐transparent part and transmitting it to the lower part, where it is converted into thermal energy, leading to the melting of resin at the interface and the entanglement of resin chains to create a joint as a result. The laser transmission welding process is heavily influenced by laser radiation, welding rate, and pressure, and it has a narrow process window.

For the CFRT parts, no laser absorber is usually required due to the laser radiation absorption of the thermoplastic matrix. Thus, in the aviation industry, the LW technique has been widely applied in joining the CFRT and metal parts, and many studies have confirmed that the mechanical interlocking, chemical bonding, and physical bonding can be formed between CFRT and metal parts during LW process.^[^ ^147^ ^]^ Notably, mechanical interlocking plays a dominant role in welding strength between CFRT and metal parts, and thus the surface treatment is necessary for metal parts before LW. The strength of chemical bonding is much higher than that of mechanical interlocking, so forming strong chemical bonding by surface treatment is an important development direction for the LW of CFRT and metal parts. In addition, the LW of two CFRT parts is still a great challenge because the carbon fibers can absorb and reflect the laser beamline.^[^ ^148^ ^]^

### 5.5 Coconsolidation

Coconsolidation is another effective joining method for CFRT parts. Coconsolidation implies simultaneous application of temperature and pressure to melt preforms together into one part.^[^ ^149^ ^]^ Pressure and temperature are the two main variables of a fusion bonding process.^[^ ^150^ ^]^ A seamless transition is achieved by matching the joining strength of the bulk material, minimizing cycle time and eliminating the need for additional joining materials.^[^ ^151^ ^]^ Consolidation in fusion bonding requires polymer–polymer interface healing. This is achieved in five sequential stages of surface rearrangement, surface approach, wetting, diffusion, and randomization. In addition to surface roughness, polymer temperature, and pressure, time takes to reach a tight joining can be attributed to the viscosity of the polymer. Once achieving tight joining, the interface can no longer be distinguished from the bulk material, and pressurized cooling completes the coconsolidation. The cooling rate also affects both structural and mechanical properties of the joined CFRT parts.^[^ ^151^ ^]^

Coconsolidation greatly reduces the amount of assembly work and therefore the production cost. Coconsolidation has been widely applied in carbon fiber reinforced thermoset composite, but the coconsolidation of CFRT parts needs further exploration due to the high viscosities (100–1000 Pa s) and processing temperatures (380 °C) of the thermoplastic matrices.^[^ ^149^ ^]^ Obviously, the co‐consolidation process parameters of CFRT parts need to be further optimized through systematic research, and the software simulations should be applied.

## 6 CFRTs Applications

CFRTs are increasingly being utilized in novel applications in a range of established and emerging sectors including aerospace, automotive, and gas storage in pristine and recycled forms. In aerospace, the CFRT parts boost fuel efficiency and performance by making aircraft lighter. In the automotive sector, these materials enhance vehicle safety and fuel economy. For pressure vessels, they provide exceptional durability and withstand extreme conditions. Moreover, the recyclability of thermoplastics in CFRT parts helps address environmental concerns, making them a sustainable option for many uses. Intermediate forms of continuous‐fiber CFRTs are predominantly used in for aerospace, automotive, and pressure vessels specifically using TTW, whereas discontinuous LCFTs and short fiber‐based CFRTs are used for high volume automotive parts.

### 6.1 Aerospace

Aerospace applications have witnessed the growth and resurgence of CFRTs as effective alternatives for carbon‐fiber‐reinforced thermosets and metallic components because of their higher impact resistance, versatile processing, light weight, and environmental credentials (the potential of recyclability).^[^ ^19^ ^]^ With fuel efficiency being a key driver in aerospace, CFRTs offer enormous potential for future usage because of their key attributes of weldability, recyclability, and compatibility with metallic components, which broaden their utilization possibilities. Established CFRT applications in the aerospace sector are shown in **Table** **2**, which demonstrates the efficacy of their potential. One such area is the advanced aerial mobility sector that includes uncrewed aerial applications, which potentially require weight reduction, strength and durability, efficiency in manufacturing, and recyclability to meet the future needs of the sector. For instance, PEI‐based CFRTs are widely used in aerospace interiors (e.g., seat shells, ducting channels, galleys, and trolleys) due to excellent flame smoke toxicity (FST) properties and certain chemical resistance. PPS‐based CFRTs are widely used on primary and secondary structures and aircraft interiors. PEEK‐based CFRTs are used on primary and secondary aircraft structures and interior structural components, which is similar for LMPAEK‐ and PEKK‐based CFRTs.

**Table 2:** A list of CFRT applications in aerospace based on the polymer matrix.^[^ ^152^ ^]^

Polymer	Aircraft Platform	Applications	Polymer	Aircraft Platform	Applications
PEKK	RC‐135	Radomes	PEI	Boeing 737	Smoke detector pass
PEEK	A320	Vertical stabilizer brackets		Boeing 737/757	Galleys
	EH101	Helicopter floor		Boeing 747	Stowage hims
	F‐117	Rudder assembly		Boeing 767	Acoustical tile
	F‐22	Weapons bay doors, access covers			Brackets
				Airbus A320	Cargo floor sandwich panels
	OH‐58D	Helicopter horizontal stabilizer		Airbus Beluga	Entrance floor panel
	Rafale	Engine tunnels		Dornier 328	Landing flap ribs
PPS	Airbus A330	Rudder nose ribs			Ice protection plates
	Airbus A340	Aileron ribs Fixed‐wing leading‐edge assemblies, wing access panch, beam ribs, and beam connecting angles		Fokker 50	Ice protection plates Trailing‐edge wing shroud skins
				Fokker 70/100	Structural floor panels
		Pylon pancis		Gulfstream	Structural floor pancis
	Fokker 50	Main landing gear door			Rudder ribs and trailing edges

**Figure 10:**
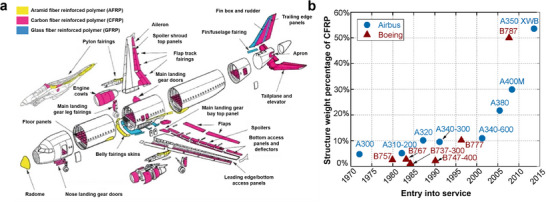
a) Illustration of aircraft showing CFRP components. Adapted with permission.^[^ ^156^ ^]^ Copyright 2023, Springer Nature. b) Illustration of continually increasing weight percentage of CFRP parts in aircraft. Adapted with permission.^[^ ^2^ ^]^ Copyright 2023, Elsevier.

The application of CFRT parts in aircraft has gradually developed over the past several decades. Initially, the CFRTs are used in the production of small components in aerospace, and the development of advanced manufacturing technologies allowed their applications in large‐scale parts.^[^ ^153^ ^]^ Often, key components manufactured using efficient one‐step consolidation such as press formed ribs and welded assemblies endow CFRTs with an distinct advantage over carbon‐fiber‐reinforced thermosets.^[^ ^154^ ^]^ Single or multiply CFRTs preforms are processed using batch or semicontinuous processes including stamp forming, AFP, and ATP, and assembled using UW and RW, which results in significant cost and weight savings in comparison to carbon‐fiber‐reinforced thermoset components, as demonstrated by Fokker.^[^ ^155^ ^]^ These incredible achievements are further enhanced through the implementation of one‐step coconsolidation of prepreg and injection‐molded sections. The aerospace sector is set to further gain from these advancements in the future, as exemplified by the multifunctional fuselage demonstrator. **Figure** **10**a presents the major CFRP parts in aircraft, and Figure 10b demonstrates that the CFRP components are increasingly being used in aircraft.

#### 6.1.1 Multifunctional Fuselage Demonstrator

Multifunctional fuselage demonstrator (MFFD) is an initiative on creating a fully thermoplastic fuselage structure for future aircraft platforms by integrating cabin, hydraulic, electrical systems, and structural components. Since the early 1980s, composite parts have been used in planes, but they have only recently been used in complete fuselages and wings.^[^ ^127^ ^]^ The MFFD project aims to overcome the major component assembly (MCA) and final assembly line (FAL) bottlenecks that limit production rates by parallelizing manufacturing, installation, and assembly processes, thereby increasing efficiency and reducing costs. The main goals of the MFFD project include achieving a high production rate of 70–100 aircraft per month, reducing recurring costs by €1 million per fuselage and reducing the fuselage structure weight by 1000 kg compared to the A321 ACF.

**Figure 11:**
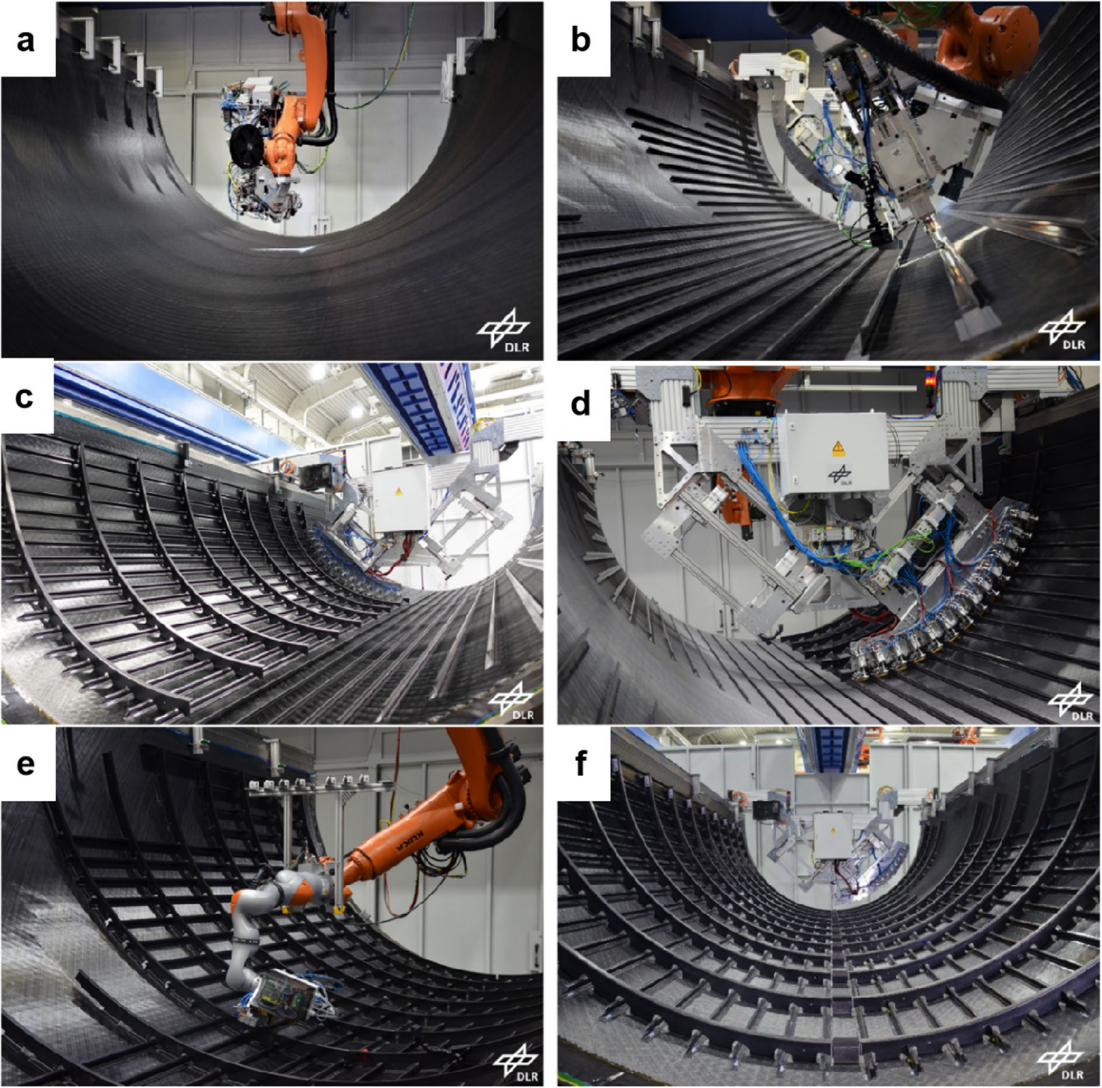
Production steps of MFFD: a) lay‐up of skin, b) continuous UW of stringers, c) RW of frames with weld bridge, d) weld bridge from the other side, e) cleat integration, and f) final upper‐shell demonstrator. Reproduced under the terms of the CC‐BY Creative Commons Attribution 4.0 International license (https://creativecommons.org/licenses/by/4.0).^[^ ^157^ ^]^ Copyright 2025, The Authors, published by Elsevier.

As presented in **Figure** **11**, the MFFD utilizes CFRTs and advanced manufacturing techniques to construct the fuselage, including AFP by laying down and consolidating UD tape, eliminating the need for additional vacuum bagging or autoclave curing, continuous UW to join stringers to the fuselage skin, ensuring strong, reliable bonds without generating debris, and RW for integrating frames and cleats, providing robust mechanical performance and reproducibility.

The upper half of the fuselage was produced by the German Aerospace Center (DLR) in three essential steps of creating skin, integrating stringers, and integrating frames and cleats. These steps involved the use of AFP to lay down tape, aligning and welding stringers using robotic UW, and using RW to attach the frames and cleats (Figure 11). The lower half was developed under the STUNNING project under the leadership of GKN Fokker. The steps in the lower half production were manufacturing of skin and assembly of stringer/frame. These steps involved the use of AFP to create perpendicular sections being consolidated into a 180° skin, integrating omega stringers and UW of frames.

The assembly of the fuselage involved the joining of the upper and lower shells using specialized tools and advanced software control capabilities, which is surmised to be further enhanced using the European MultiFAL project aims to develop an automated system for this purpose, ensuring efficient and accurate assembly.

The lower half of MFFD is a resultant of high‐TRL advancements and structural integration of fuselage panels, sub‐structure, floor beams, cabin parts, and cargo doors. AFP was used for an 8.5‐m by‐4‐m‐diameter skin lay‐up, consolidated in an autoclave, and assembled/integrated. The assembly of lower and upper halves utilize a butt‐strap longitudinal fuselage joint, on one side and a fuselage joint completed on the opposing side using UW. A fixture holds the shells together during joining. Advanced assistance for the placement of the halves is achieved through force and torque sensors further guided by optical systems. No further postprocessing is needed, but instead of applying a single ply of tape, 6‐ply fully consolidated laminates are used. A CO_2_ laser instead of the fiber lasers typical for AFP is applied, since LMPAEK polymer shows almost no energy absorption at the 1060‐nanometer wavelengths normally used by those lasers. A CO_2_ laser with a 10.6‐micrometer wavelength to ensure better heating of the polymer matrix at the surface with less migration through the laminate plies is used. An AFP end effector places three 0.5‐inch‐wide tows with a possible throughput up to 4.4 kg h^−1^ for a minimum production time of 32 h.^[^ ^158^ ^]^ Hence, the MFFD showcases the extent of the capabilities of key CFRT technologies to achieve a practical breakthrough in aircraft manufacture.

A deeper analysis of the MFFD project reveals the assembly of the fuselage involved the joining of the upper and lower shells using specialized tools and advanced software control capabilities (**Table** **3**), which is surmised to be further enhanced using the European MultiFAL project aiming to develop an automated system for this purpose, ensuring efficient and accurate assembly. AFP provided reduced lead times and improved manufacturing efficiency, although the process requires precise control of parameters to ensure quality. UW demonstrated robustness and repeatability, with room for improvements for consistency. Similarly, current leakage issues in RW need to be addressed. Extensive process development at the coupon level was necessary before scaling up to full‐scale production. This included detailed root cause failure analysis to understand and mitigate risks. Adapting technologies like RW and UW to an industrial scale required significant effort. These technologies had to be refined and optimized for large‐scale applications. The project involved collaboration between multiple partners, each contributing different technologies and expertise. Integrating these contributions into a cohesive assembly process was challenging but essential for success.

**Table 3:** A list of the key projects, their deliverables, leading organization, and partners in the aerospace.

Project	Main Deliverable (s)	Industry Leader	Industry Partners
STUNNING	Lower fuselage shell	GKN Fokker	Diehl, NLR, SAM XL, TU Delft
ECO ‐CLP	Injection molded clips, brackets recycled from	GKN Fokker	Almen, Altip
EMOTION	Consolidation tool for skin	NLR	Alpex, Ostseestaal, TU Munich
MECATESTERS	Characterization of CF/PAEK welded joints,	GKN Fokker	KVE, Rescoll
	Surfaces, process		
MISSION	Simplified hardware, cabling	GKN Fokker	Aeromechs, HSLU
TCTool	Assembly cradle, multi ‐welding end effector,	GKN Fokker	Acroflight, Brunel Univ.
	Digital twin		FADA ‐Catec, LSBU, TWI
TORNADO	Disbond arrest features	GKN Fokker	KVE, Rescoll, UPAT
	Omega stringers and cargo floor beams using	Xells	
	Cargo door surround structure (DSS)	Aernnova	CETMA, Techni ‐Modul
	Upper fuselage shell	DLR ZLP Augsburg	Premium Aerotec
	Compression molded Z ‐stringers	Aernnova	CETMA, Techni ‐Modul
	Fuselage C ‐frames, passenger DSS, trimming	Premium Aerotec	
MultFAL	Assembly plant, lower cradle, upper hexapods	Fraunhofer	Almen, FFT, CT Engineering Group
BUSTI	LH butt ‐strap fuselage joint	Airbus	Aernnova, Almen, CTI Systems, FFT
WELDER	RH overlap longitudinal fuselage joint	Airbus	Almen, FFT
MAYA	Thermoplastic composite lining panels	Diehl Aviation	Alpex, CT ‐IPC, Leitat, University of Girona
INTELLICONT	Composite air cargo container with integrated functions	Airbus	Acciona, Avionics Greece, Prisma

Further research into CFRTs to enhance their properties, such as strength, durability, and recyclability, is essential. This includes exploring new formulations and manufacturing techniques to improve performance and reduce costs. The MFFD project represents a significant advancement in aircraft manufacturing, demonstrating the potential of CFRTs to revolutionize the industry. The project achieved substantial weight and cost reductions paving the way for more efficient and sustainable aircraft production.

### 6.2 Composite Pressure Vessels

The global demand for CFRP‐based composite pressure vessels (CPVs) for gas and liquid storage amounts to 8% and there are plans in the carbon fiber production industry to boost capacity by more than 20% by 2025.^[^ ^1^ ^]^ Compressed gas storage today is a market that is dominated by CFRPs, with cost and technology barriers such as high manufacturing cost, energy intensive, long curing, and little to no recyclability.^[^ ^159^, ^160^ ^]^ As established, CFRTs are a reliable alternative for carbon‐fiber‐reinforced thermosets in other sectors, and this is true for CPVs as well, albeit CFRTs suffer from a delayed start and relative inexperience in design and development compared to carbon‐fiber‐reinforced thermosets.^[^ ^161^ ^]^ CFRT tape intermediates are used to lay up or wind pressure vessels using AFP and TTW processing methods, respectively. CPV development today is aimed at catering to the fuel cell electric vehicle energy (H_2_) storage. Although H_2_ is a low‐density gas, its energy density per unit mass is much higher than conventional fossil fuels. Permeation resistance in CPVs is a significant concern and the fuel dissipation and loss mean achieving a 500 km‐per‐tank range is challenging without adding vehicle mass for onboard CPV storage.^[^ ^162^ ^]^ The UN134 regulation deems a working pressure of 20–70 MPa and burst strength with at least safety factor of 2 is necessary to safely and effectively use CFRT CPVs. Trucks, cars and trains are predicted to use conformable onboard H_2_ CPVs in the future.^[^ ^163^ ^]^

**Figure 12:**
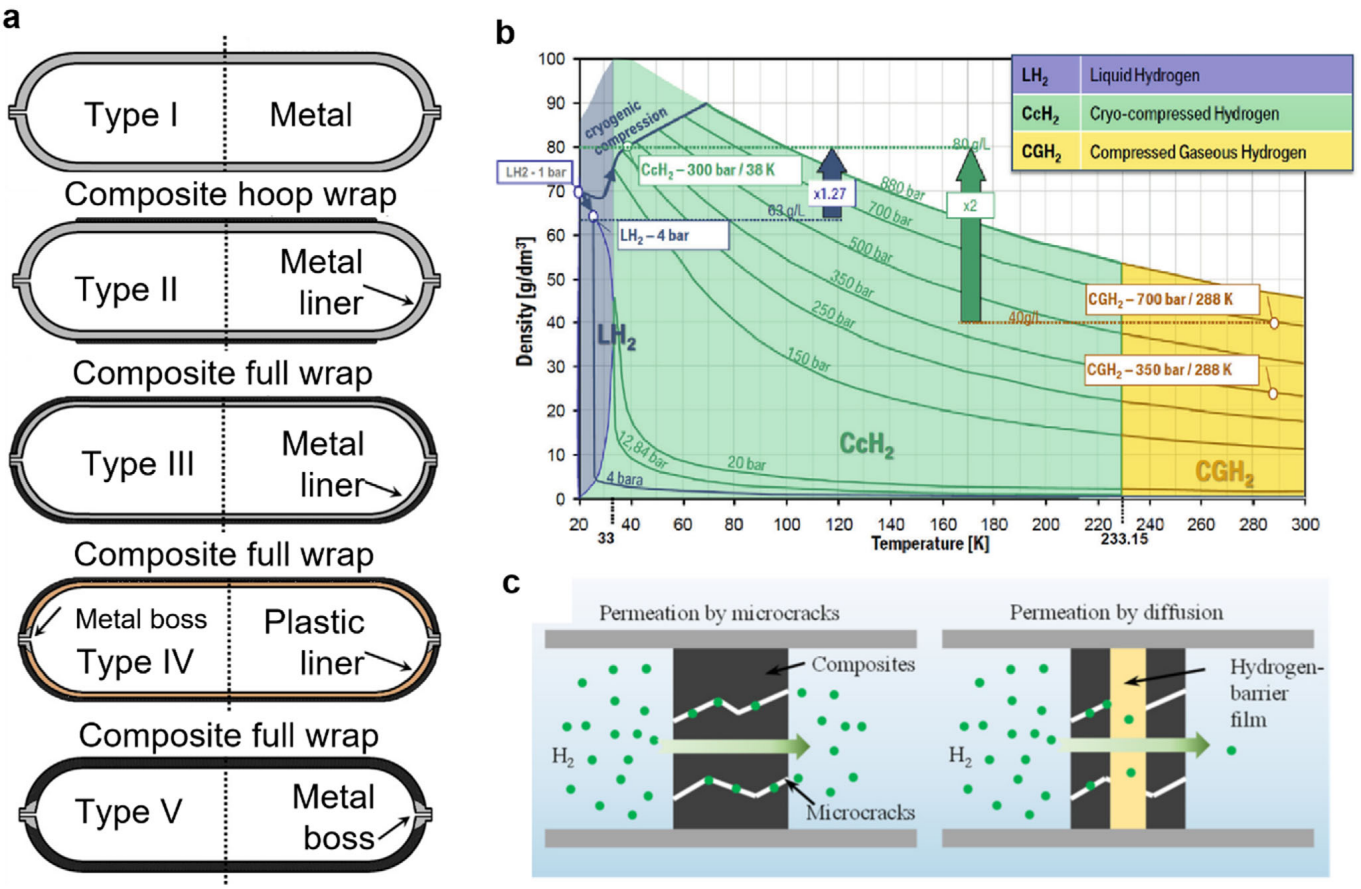
a) Schematic of various CPV designs based on construction. Adapted with permission.^[^ ^166^ ^]^ Copyright 2025, Elsevier. b) Storage density versus system temperature, and c) susceptibility of the wall or liner materials to hydrogen permeation and diffusion leading to microcracking in the matrix along fiber peripheries. Adapted with permission.^[^ ^163^ ^]^ Copyright 2023, Elsevier.

Hydrogen can be stored safely and efficiently in form of cryo‐compressed hydrogen (CcH_2_), compressed gaseous hydrogen (CGH_2_), and liquid hydrogen (LH_2_) (**Figure** **12**b).^[^ ^163^ ^]^ CcH_2_ stores hydrogen at low temperature (20–50 K) and high pressure (35 MPa), provides an optimal solution to H_2_ storage. Take for example a single aisle aircraft, where the energy density only approaches as viable only at cryo‐compressed condition.^[^ ^160^, ^164^ ^]^ However, this brings in the conundrum of designing embrittlement and permeation resistance for the CFRTs.

To address the demanding conditions of H_2_ storage, four different types of pressure vessels are developed, as shown in Figure 12a. They range from an entire metallic construction to an entirely composite construction, with the intermediate types offering some form of combination of both. As the designs progress from Type I to Type V, overall mass (density) decreases, with Type I being the heaviest yet the most cost‐effective, and vice versa. Fatigue resistance and volumetric capacity progressively improve from Type I to Type V as well.^[^ ^163^ ^]^ The CPV type also dictates the permeation and crack initiation and progression mechanisms through the CPV wall. The material of the structure should resist microcracking and permeating of H_2_ through the laminate (Figure 12c). Microcracking on the surface leads to hydrogen permeation. A homogeneous laminate and material structure reduces thermal stress, resulting in improved mechanical performance. Often a polymeric barrier is used in the form of a liner, which may bring in further complexities in terms of strain incompatibility, especially in the hoop or overwrapped Type II–IV constructions. Carbon fiber is commonly used to support part or all of the structural load in type II, III, and IV vessels (Figure 12a). Due to their light weight and high storage density, the Type IV vessels are often used in ground transport applications, with notable examples being Toyota Mirai and Honda Clarity that use 70 MPa CPVs. No internal liner is used in Type V, which is a pure composite construction where the composite acts as a load bearing structure and a gas barrier. The lack of a liner eliminates the need for strain compatibility between the liner and composite, which increases fatigue resistance and reduces weight by 10–20%.^[^ ^165^ ^]^

CFRT tapes used in CPVs are fabricated starting from polymer treatment and then creating a mixture with the UD fibers, which are wound or laid on a mandrel for Types with liners. When AFP or TTW are used, the consolidation of preform occurs under pressure at an elevated temperature beyond the *T*_m_ of the polymer to create a densely consolidated CFRT network. The overall process parameters of significance are temperature, roll pressure, layup speed, and tension in the tape. Thermoplastic matrix dictates the thermomechanical performance of the vessel, where drastic temperature during filling cycle leads to thermomechanical stress in the vessel walls. It has been proven that thermomechanical fatigue damage occurs more easily in composites when loaded thermally and mechanically, with only a few cycles required to initiate the failure.^[^ ^165^ ^]^ Conversely, cryogenic storage leads to fiber contraction ultimately reducing the fiber strength and compromise its fatigue performance.^[^ ^167^ ^]^ Degradation arising from thermomechanical stresses is an issue for CFRPs.

CPVs processed using AFP require a near zero porosity tape with high alignment of fibers, and preferably a high‐volume fraction of carbon fibers. This ensures high modulus and strength at a lower unit mass of the construction. In terms of the polymer matrix, a molten polymer is preferred versus a powder that may require dissolution into a solvent. The use of polymer films is also a viable method of ensuring that the necessary property range is achieved, albeit this might require a primary processing method such as sheet formation, which adds to cost. Nonetheless, a CFRT vessel offers improved permeability resistance and the versatility to integrated with the balance of plant equipment via welding as opposed to bolted joints.

CFRTs for CPVs have been investigated by major research consortiums across the world, such as the Institute for Advanced Composites Manufacturing Innovation (IACMI) and The Netherlands (NL) LH_2_ composite tank consortium.^[^ ^168^ ^]^ The IACMI consortium focused on 70 MPa Type III tank manufactured using UD CFRT tape, and the NL consortium focused on producing Type V LH_2_ vessels for single aisle aircraft.^[^ ^169^ ^]^

### 6.3 Automotive

CFRT applications in the automotive sector are aimed at driving down CO_2_ emissions, with the advent of electric vehicles requiring greater weight reduction. Additionally, the demand for recyclability and affordability because of the large volume manufacturing has led to the positioning of CFRTs with engineering thermoplastics such as PA and PP to be adopted with cycle times ranging to a few min.^[^ ^170^ ^]^ CFRTs based on PA are used in exterior and interior automotive parts, e.g., door modules, body‐in‐white components, steering wheel, seats, exterior mirrors, front‐end grilles, and trim.

Carbon‐fiber‐reinforced thermosets are used in automotive parts with demonstrated success, albeit their long processing cycles have rendered them uncompetitive especially in comparison to CFRTs. This contrast has prompted significant technological advancements in the field.^[^ ^20^ ^]^ Discontinuous carbon fiber based CFRTs such as short carbon fiber based CFRTs and LCFTs have gained a significant application share in automotives. CFRTs originally developed for aerospace using high‐performance thermoplastics such as PEEK face major limitations in adapting to new requirements. Despite their advantages, their high cost remains a major obstacle. Additionally, the brittleness of semicrystalline CFRTs limits their feasibility in the automotive industry, where materials need high energy absorption capabilities.^[^ ^20^ ^]^

PP and PA based CFRTs are therefore well‐suited for the automotive industry. While continuous carbon fiber based CFRTs struggle with forming complex shapes, discontinuous carbon fiber based CFRTs, e.g., processed using injection molding and LCFT‐D, often fall short in meeting the mechanical requirements for primary structural parts in vehicles. To overcome these challenges, new specialized discontinuous CFRT intermediate materials have been developed. These semifinished and semiimpregnated products maintain the linearity and sufficient aspect ratio of carbon fibers, providing high performance, affordability, formability, and recyclability.^[^ ^20^, ^64^ ^]^

Various CFRT intermediates are being developed to increase the effectiveness of carbon fiber, e.g., chopped tape, mat‐reinforced thermoplastics, paper‐reinforced thermoplastics, and card web‐reinforced thermoplastics. These intermediates demonstrate higher functionality because of the feasibility to design lightweight lattices, sandwich structures, and orientated fiber alignment hence providing flexible application opportunities.^[^ ^20^ ^]^

Chopped tape CFRTs with a PA6 matrix exhibit similar mechanical properties to continuous CFRPs in terms of maximum tensile and flexural properties, indicating mechanical properties depend on tape thickness and fabrication. Ultra‐thin prepregs used in chopped tape enhance mechanical properties supporting the design of a fabrication process ideal for mass production. Compared to conventional products, the reduced tape thickness allows for 10 times faster resin impregnation. Since the tape is small, a low‐cost papermaking method is used for dispersion, thereby considerably reducing the manufacturing cost and time of prepregs and components, which is ideal for mass production in the automotive industry.^[^ ^20^ ^]^ However, this comes with the need to optimize tape distribution and microstructure. Workability with longer tapes and thinner prepregs has known advantages, but also prolonging the processing cycle, driving up component cost, and necessitating careful consideration to achieve an application‐specific balance in mechanical properties, tape structure, and processing.^[^ ^20^ ^]^

CFRT manufacturing cost analysis is crucial for assessing the feasibility of corresponding research and development items to reduce overall costs in the automotive industry since cost is a major driver. These costs can be classified as: i) carbon fiber, ii) matrix resin, iii) prepreg fabrication, and iv) molding.^[^ ^20^ ^]^ Parts that have a high cost‐sensitivity are those with a high molding cycle time, meaning that reducing molding cycle time can effectively reduce part costs. A significant cost difference is also caused by carbon fiber cost; but innovation in carbon fiber is necessary to significantly lower carbon fiber costs. It is possible to increase the effective utilization ratio of carbon fiber by recycling waste carbon fiber directly in the manufacturing plant. Further, CFRT efficiency will be increased by reducing the amount of carbon fiber used in a structure/material design. With CFRT, costs can be reduced to the level of aluminum alloys, providing a solid foundation for mass use in vehicles.^[^ ^20^ ^]^

The use of thin and ultrathin tapes is prevalent in the automotive sector. The HybCar project uses fiber placement process to lay up UD CFRT tapes on metallic substrate, with the freedom to further stiffen the section using thermoplastic pultruded profiles, resulting in structurally reduced vehicle floor height and its associated advantages in efficiency.^[^ ^171^ ^]^ A fully automated manufacturing process reduced production cycle time to under 90 s and decreased weight by over 10 kg (≈40%). Similarly, other successful examples include a bionic tape structure integrated into injection‐molded components by AZL Aachen GmbH, and overmolded carbon fiber‐PP UD tape for liftgate structure, which embodies the principle of local stiffening by using CFRTs in locations that require structural strength.

### 6.4 Recycling

**Figure 13:**
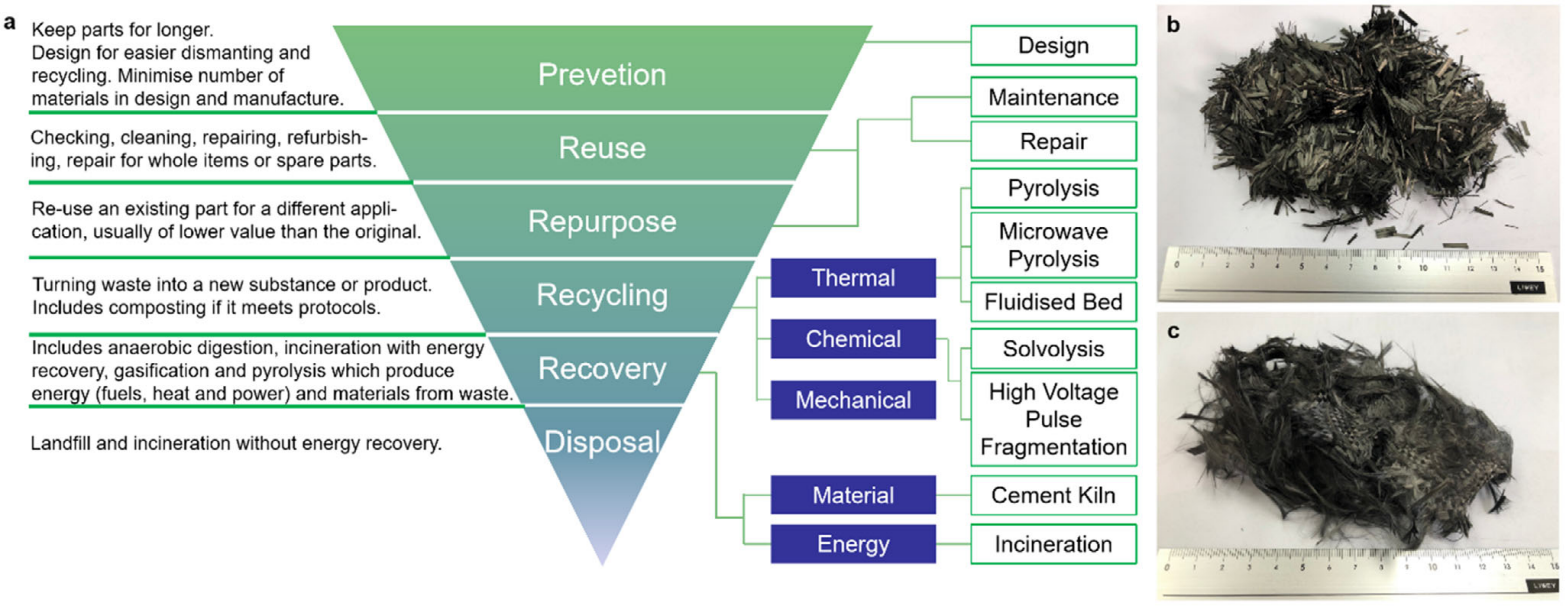
a) European waste hierarchy. Adapted with permission.^[^ ^174^ ^]^ Copyright 2023, Elsevier. b) Offcut carbon fiber, and c) recycled carbon fiber. Adapted with permission.^[^ ^175^ ^]^ Copyright 2022, Elsevier.

Recyclability has always been a strength of CFRTs, however, this has remained at a small scale and yet to be realized commercially at the large scale. Recycling technologies play a crucial role in making carbon fiber composites more sustainable and cost‐effective (**Figure** **13**).^[^ ^172^ ^]^ By effectively utilizing strong in‐plane anisotropy through object‐oriented structure optimization, these materials offer flexible options for various applications.^[^ ^173^ ^]^ This approach not only improves performance but also supports environmental sustainability by promoting the recycling of carbon fiber composites. A major example of recycling is the MFFD where using injection molding short fiber blends is carried out from scrap by recycling.

In 2016, TenCate Advanced Composites and the Thermoplastic Composites Research Center collaborated with GKN Fokker to showcase the benefits of recycling in the CFRP field. During the production of the Gulfstream G650 elevator and rudder, GKN Fokker generated scrap material from TenCate Cetex TC1100 woven carbon fiber/PPS. This scrap was repurposed to develop an access door panel. This demonstrated use of reclaimed material enables lightweight parts and allows parts to achieve geometric shapes that would otherwise be impossible. By applying a flow layer of recycled material, stiffening ribs, and variable thicknesses that can be molded into the part, along with bosses around holes that distribute load generated by fasteners. As a result, less continuous fiber material is needed, resulting in thinner, lighter, and more cost‐effective parts compared with those made from virgin materials alone.^[^ ^176^ ^]^ Similarly, a method of foam‐based forming akin to thermoplastic foam injection molding where a blowing agent was added to a melt and injection molded. The inclusion of a low carbon fiber content aided with cell nucleation and promoted foam formation. Using this processing method, complex sandwich structures with varying wall thickness and curvature were produced using recycled carbon fibers infused with PA.^[^ ^111^ ^]^

By integrating recycled carbon fibers into production, manufacturers can reduce waste and lower costs while maintaining high performance standards.^[^ ^177^ ^]^ This method is particularly beneficial in industries such as automotive and aerospace, where weight reduction and material efficiency are critical.^[^ ^178^ ^]^ Moreover, the environmental benefits of using recycled carbon fibers extend beyond the manufacturing process.^[^ ^179^ ^]^ The high value and versatility of recycled carbon fiber composites allows for innovative applications across various sectors.^[^ ^1^, ^33^, ^180^ ^]^

## 7 Conclusion and Future Perspectives

The advancement of automated processes such as AFP and ATL is pivotal for the future of CFRTs. These technologies facilitate in situ consolidation, significantly reducing cycle times and energy consumption. Future developments will likely enhance the speed and precision of these systems, making CFRTs more cost‐effective and scalable for large‐scale production. The aerospace sector requires affordable, automated, and rapid processes for manufacturing complex CFRT structures. Innovative and effective joining techniques are also becoming essential. Fiber steering, a novel feature in AFP, optimizes fiber orientation for complex shapes, surpassing the traditional 0°/45°/90° orientations. Additionally, OOA forming and coconsolidation technologies are being extended to more challenging applications, such as complex spars, beams, and integrally stiffened skins.

The development of new thermoplastic matrices and improved prepreg materials contribute to enhancing the performance of CFRTs. Innovations in semicrystalline polymers like PEEK and PEKK are particularly noteworthy. Future research will likely explore hybrid materials that combine the benefits of different polymers and nanomaterials to further enhance properties such as electrical conductivity and impact resistance. There is a growing demand for thicker tapes, with a thickness of up to 0.18 mm, and optimizing tape quality for higher‐quality parts at faster speeds is crucial. Further improvements can be achieved through the integration of local reinforcements and metal brackets. Hybrid semicrystalline/amorphous polymers present another solution, with new concepts developed to maintain the advantages of working in the amorphous state without affecting crystallinity. Meanwhile, effective surface treatments of carbon fibers should also be paid attention to.

CFRTs offer significant environmental benefits, including recyclability and reduced waste. The ability of CFRTs to remelt and reform without significant loss of their properties makes them ideal for sustainable manufacturing practices. Future developments will be focused on improving recycling processes and expanding the use of recycled CFRTs in high‐performance applications.

The aerospace sector will continue to drive CFRT development with innovations such as organosheet, with applications expanding from secondary structures to primary load‐bearing components. The automotive industry is also expected to increase its use of CFRTs for lightweight, durable parts. Future research will likely address challenges in joining techniques, such as welding and fusion bonding, to ensure reliable and efficient assembly of complex structures. Developments will focus on vacuum and press consolidation, size scaling, and adapting to curved contours. In situ process monitoring, inspection, and control are also critical, with advancements in CFRT repair, and nonaerospace applications.

Despite their advantages, CFRTs face challenges such as high processing temperatures and the need for specialized equipment. Future research will be focused on overcoming these barriers by developing new processing techniques and improving the understanding of the relationship between processing parameters and material properties.

## Conflict of Interest

The authors declare no conflict of interest.
